# Integrating Machine Learning and Transcriptomics to Enhance β‑Carotene Production in *Saccharomyces cerevisiae*

**DOI:** 10.1021/acsomega.6c06012

**Published:** 2026-08-11

**Authors:** Peerapat Khamwachirapithak, Kittapong Sae-Tang, Suriyaporn Bubphasawan, Jongrak Choompuper, Xin-Qing Zhao, Verawat Champreda, Weerawat Runguphan

**Affiliations:** † Department of Biotechnology, Faculty of Science and Technology, Rangsit Campus, 37698 Thammasat University, Phahonyothin Road, Khlong Luang, Bangkok, Pathum Thani 12120, Thailand; ‡ National Center for Genetic Engineering and Biotechnology (BIOTEC), National Science and Technology Development Agency (NSTDA), 111 Thailand Science Park, Phahonyothin Road, Khlong Nueng, Khlong Luang, Bangkok, Pathum Thani 12120, Thailand; § State Key Laboratory of Microbial Metabolism, Joint International Research Laboratory of Metabolic & DevelopmentalSciences, School of Life Sciences and Biotechnology, 12474 Shanghai Jiao Tong University, Shanghai 200240, People’s Republic of China; ∥ Industrial Bioprocess Innovation Group, The Eastern Economic Corridor of Innovation, National Science and Technology Development Agency (NSTDA), 333 EECi Headquarters Wang Chan, Rayong 21210, Thailand

## Abstract

Optimization of microbial production by synthetic biology is essential for industrial and sustainable biotechnology applications. β-carotene is a high-value compound that can be heterologously produced in the budding yeast *Saccharomyces cerevisiae*, providing an alternative to natural extraction. In this study, we aimed to enhance β-carotene production by machine learning–guided combinatorial strain engineering and transcriptomic analyses. We fine-tuned the expression of rate-limiting enzymes in the mevalonate (MVA) pathway through combinatorial engineering of promoters and terminators. XGBoost was applied to the Design-Build-Test-Learn (DBTL) cycle to facilitate rapid optimization. In the second DBTL cycle of 1 mL culture screening, fine-tuning MVA gene expression resulted in a 139% improvement in β-carotene titer. Additionally, guided by transcriptomic insights into altered expression of iron uptake genes, we supplemented β-carotene production cultures with iron, resulting in a 70.54% increase in β-carotene titer. Furthermore, integrating the fine-tuned MVA cassette with iron supplementation in 250 mL shake-flasks yielded up to 72.07 mg/L of β-carotene at 72 h, representing a 67.79% increase compared to the β-carotene-producing strain without MVA gene fine-tuning. Our study demonstrates the effectiveness of XGBoost in predicting complex combinatorial designs and highlights the potential of combining machine learning and transcriptomic insights to optimize non-native biochemical production in yeast.

## Graphical abstract

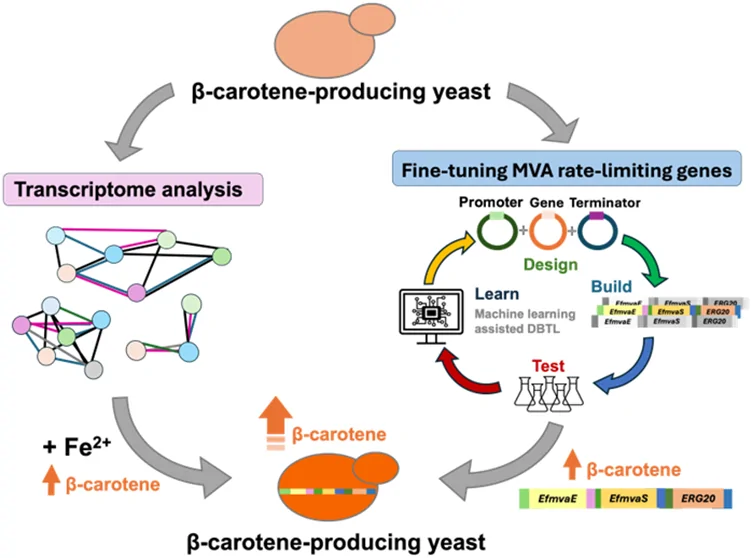

## Introduction

Microbial production has enabled large-scale production of food, beverages, and biobased products since its early applications in alcoholic drinks and fermented dairy products. Recently, with increasing advances in biotechnology research, microbial production using yeasts recognized as Generally Recognized as Safe (GRAS) strains, such as *Saccharomyces cerevisiae* and *Pichia pastoris*, has been extensively utilized in nutraceutical, pharmaceutical, and bioindustrial applications.^1^ *S. cerevisiae*, commonly known as the baker’s yeast, has been widely used in brewing, bioethanol production,^2,3^ the production of recombinant proteins,^4^ and the synthesis of heterologous biochemicals such as organic acids e.g., lactic acid^5^ and other high-value metabolites through genetic and metabolic engineering.^6–8^ Moreover, yeast production of high-value compounds offers a more sustainable alternative to traditional chemical synthesis or fossil fuel–based processes.^9,10^ Although metabolic engineering and synthetic biology studies have improved the production of non-native compounds in *S. cerevisiae* through heterologous expression or laboratory evolution, significant challenges remain. Further advances will require the integration of emerging technologies, such as next-generation sequencing and computational modeling, to better understand and reprogram complex microbial regulatory networks.

Carotenoids, including lycopene, β-carotene, and astaxanthin, are high-value natural compounds widely used in the food, cosmetics, and pharmaceutical industries due to their antioxidant properties and health benefits.^11,12^ The growing demand for carotenoids has spurred studies in metabolic engineering and synthetic biology, as microbial fermentation offers a viable alternative to extraction from plants or other natural sources. Several studies have demonstrated the heterologous expression of carotenogenic genes from *Xanthophyllomyces dendrorhous* and *Sporidiobolus pararoseus* in yeast.^6,8,13,14^ Overexpression of rate-limiting enzymes is a common strategy employed in many bioproduction platforms, including violacein^15^ and β-carotene.^16^ The mevalonate (MVA) pathway, which generates the initial precursor supply for carotenoid biosynthesis, has demonstrated promise as a metabolic target for optimizing carotenogenic microbes^17^ and enhancing sesquiterpene production.^18^ Increasing the expression of MVA enzymes has been shown to improve carotenoid titers through both adaptive laboratory evolution^19^ and targeted gene overexpression.^8,17^ Unlike yeast, bacteria naturally lack the MVA pathway and instead rely on the methylerythritol 4-phosphate (MEP) pathway to produce carotenoid precursors, thus requiring additional metabolic engineering efforts.^20^ Consequently, studies focusing on the MVA pathway in yeast have identified key candidate genes for improving carotenoid production.^21–23^

The rapid development of artificial intelligence-based tools and technologies has enhanced and accelerated complex studies in synthetic biology. For example, machine learning employing probabilistic ensemble techniques has guided the optimization of limonene and dodecanol production.^24^ Additionally, gradient-boosted tree–based algorithms such as XGBoost have been used to optimize ethanol production via promoter modifications.^25^ In another case, gene expression levels and D-lactic acid production were correlated using predicted transcriptome profiles, leading to increased D-lactic acid production.^26^ Moreover, artificial neural networks (ANNs) have improved production of β-carotene and violacein production,^27^ as well as lycopene^28^ and limonene,^29^ via multilayer perceptron. Furthermore, advances in genomics and transcriptomics, enabled by next-generation sequencing, have provided a deeper understanding of molecular responses and the fundamental knowledge required for microbial engineering and production. For instance, a genomic comparison revealed three gene targets involved in astaxanthin bioproduction, leading to increased biosynthesis.^7^ Similarly, transcriptome analysis of β-carotene-producing strains from laboratory evolution revealed upregulation of lipid biosynthesis and the MVA pathway.^19^ Collectively, integrative analytical approaches, including transcriptomic sequencing and machine learning, offer powerful approaches to optimize engineered microbial strains for both academic and industrial applications.

In this study, we aimed to optimize β-carotene production in *S. cerevisiae* by combining synthetic biology with machine learning and systems biology approaches. Specifically, we employed XGBoost, a gradient boosting algorithm, to guide combinatorial fine-tuning of promoter–terminator combinations for both native and non-native rate-limiting genes in the MVA pathway. This strategy yielded a 2-fold improvement in β-carotene production in the second DBTL cycle. In addition, we compared the transcriptomes of a metabolically engineered *S. cerevisiae* strain producing β-carotene with those of its parent strain. Our transcriptomic analysis revealed transcriptional responses resembling those under iron deficiency, together with upregulation of amino acid metabolism. Subsequently, we combined machine learning-driven strain optimization with iron supplementation, guided by transcriptomic insights, resulting in a further improvement in β-carotene production. Overall, our findings illustrate how integrating machine learning-guided synthetic biology with transcriptomic analysis can significantly enhance bioproduction efficiency.

## Materials and Methods

### Strain Constructions, Media, and Yeast Transformation

The parental yeast strain used for construction in this study was *S. cerevisiae* CEN.PK2–1C, with the genotype *MATa* his3Δ1 leu2–3,112 ura3–52 trp1–289 MAL2–8c SUC2. To construct the β-carotene-producing yeast, the carotenogenic genes *crtI*, *crtE*, and *crtYB*, which encode phytoene desaturase, geranylgeranyl diphosphate synthase, and phytoene synthase/lycopene cyclase, respectively, were assembled into the plasmid pRSII416-Sp-Sp-Sp harboring 1021bup-TPRM9-SpCrtE-PGAL1-PGAL10-SpCrtYB-TGAL10-PGAL7-SpCrtI-TCPS1–1021bdown and integrated into the ARS1622 locus of *S. cerevisiae* CEN.PK2–1C using uracil auxotrophic selection. The *GAL80* gene was deleted to eliminate the requirement of galactose for GAL promoter induction using the Cre/LoxP system.^8^ Deletion of *GAL8* *0*, a repressor of*GAL* gene expression, enables constitutive activation of GAL promoters even in glucose-containing media. This not only simplifies cultivation but also reduces operational cost, as galactose is substantially more expensive than glucose, making the system more practical for large-scale or cost-sensitive applications. The *URA3* selectable marker flanked by LoxP sites was PCR-amplified from the pUG72 plasmid, with primers designed to include 42-bp overhangs homologous to the upstream 5′ region and downstream 3′ region of the *GAL80* gene.^30^ Finally, the *URA3* selectable marker was excised by expressing Cre recombinase from the pSH62 plasmid, which carries the GAL1-driven *cre* gene, thereby mediating recombination between the flanking loxP sites and looping out the marker. Correct clones of β-carotene-producing yeast with Δ*gal80* were selected on YPD medium containing 1 mg/mL 5-fluoroorotic acid. The construction of β-carotene-producing yeast was previously reported.^8^ A summary of the strains used in this study is listed in Table S1. Yeast transformation was performed using the lithium acetate/single-stranded carrier DNA/PEG method as previously described.^31^ For uracil auxotrophic selection, synthetic complete medium lacking uracil was prepared with 6.7 g/L yeast nitrogen base without amino acids (Difco), 1.92 g/L uracil-dropout supplement (Formedium), and 2% glucose. The synthetic complete medium without leucine amino acid was prepared by 6.7 g/L of yeast nitrogen base without amino acids (Difco), 1.62 g/L of leucine-dropout supplement (Formedium), and 2% glucose. Primers used in this study are listed in Supporting File 1.

### RNA Extraction Followed by Next-Generation Sequencing and Analysis

Yeasts containing the carotenogenic cassette (*CrtI*, *CrtE*, and *CrtYB* genes) were cultured in YPD media with an initial optical density of 0.05 for 18 h, with experiments conducted in triplicate. RNA was extracted by RNeasy Mini Kit (Qiagen, Germany). RNA quantity and quality were determined using the Fragment analyzer (Agilent, USA) and Qubit (Thermo Fisher Scientific, USA). Sequencing libraries and sequencing were subsequently performed by BGI service (China) using the DNBSEQ stranded mRNA library and the DNBseq platform, respectively. Raw files and processed data can be found at GSE288397.

We followed the RNA-seq analysis pipeline with minor modifications described.^32^ In brief, raw reads were mapped to the sacCer3 genome using HISAT2 with “-q --no-mixed --no-discordant --dta” parameters.^33^ Duplicated reads were removed by the Picard tool.^34^ Raw read counts of transcripts were quantified using Stringtie,^35^ and subsequently identified differentially expressed genes (DEGs) by the DEseq 2 library^36^ in R programming.^37^ Three independent biological replicate transcriptomes were normalized before downstream analysis. Gene ontology (GO) was annotated by the GO Term Finder^38^ together with the Saccharomyces Genome Database.^39^ Protein–protein interaction of DEGs was analyzed using the STRING database.^40^

### Determination of Promoter and Terminator Strengths

The reporter GFPuv plasmid used in this study is described in the previous study.^25^ In brief, the GFPuv plasmid containing the *GFP* gene, TEF1 promoter, and CYC1 terminator was used for the replacement of the promoter or terminator independently for strength determination. In brief, the promoter of the GFPuv plasmid was replaced by individual 14 promoters including ENO2p, TEF2p, PGK1p, RPL3p, TIP1p, RPL15Ap, YEF3p, RPL4Ap, SSB1p, RPL8Bp, PDA1p, ADH 1p, and SSA1p. The CYC1 terminator was replaced by PRM5t, SPO1t, ADH1t, PRM9t, AIP1, MRP4t, CPS1t, GSY2t, CYC1t, HSP26t, GRE3t, ECM10t, PSY4t, and GND1t. β-carotene-producing yeasts containing different promoter and terminator plasmids were cultured in 5 mL of synthetic complete (SC) medium without uracil and 2% glucose. The incubation was 250 rpm of agitation for 24 h with three biological replicates. To determine the Green fluorescence signal produced by yeasts, the SynergyH1 microplate reader (Biotek, Aligent, USA) was used at 395 nm excitation and 509 nm emission to detect cells that resuspended in water with 1:10 dilution with triplicate.

### Combinatorial Library Construction

To construct combinatorial transcriptional units of *EfmvaE*, *EfmvaS*, and *ERG20* genes, we used the Golden Gate assembly^41^ with the YeastFab system.^42^ First, promoters and terminators were amplified using the genomic DNA of the *S. cerevisiae* CEN.PK2–1C strain and assembled into HcKan_P and HcKan_T plasmids for promoter and terminator, respectively. Heterologous genes from *Enterococcus faecalis* (*EfmvaE* and *EfmvaS*) were synthesized by GenScript (GenScript Biotech, USA), while *ERG20* was amplified from the parental genome. All genes were subsequently assembled to the HcKan_O plasmid. Combinatorial transcriptional units (cTU) were assembled by mixing 14 plasmids containing different promoters and 15 plasmids containing different terminators (HcKan_P and HcKan_T) with each HcKan_O and POT2, 4, and 5 plasmids. Golden gate assembly was carried out by NEBrigde Ligase Master Mix, BsmBI-v2, and BsaI-HF v2 (NEB, USA). Combinatorial plasmids from the reaction were directly transformed into DH5alpha competent cells and selected on LB containing 50 ug/mL kanamycin. Plasmids from all successfully assembled colonies were extracted and used in pathway assembly (cTU1: *EfmvaE*, cTU2: *EfmvaS*, cTU3: *ERG20*)

Combinatorial transcriptional units (cTU1, cTU2, and cTU3) were mixed with pMV-URR1, pMV-URR2, and pMV-LEU to construct combinatorial pathway fragments. The assembled reactions were digested by BsaI-HFv2 and ligated by NEBrigde Ligase Master Mix (NEB, USA). Next, assembled pathway fragments were transformed into β-carotene-producing yeast with linearized pRS414-URR to homologous recombination to the plasmid containing *EfmvaE*, *EfmvaS*, and *ERG20* genes with random promoter and terminator. Promoter and terminator combinations of each selected yeast colony were identified by sequencing. Finally, the selected combinatorial combination (P2–6) was integrated into an ARS308a locus in β-carotene-producing yeast and selected on an SC medium containing 2% glucose without leucine.

### High Throughput Screening and Small-Scale Production

After combinatorial construction and transformation of the MVA rate-limiting genes, combinatorial strains were cultured in an SC medium containing 2% glucose without leucine for auxotrophic selection. To enhance production experiments, the high-throughput BioLector microbioreactor (BioLectorPro, m2p-laboratories, Germany)^43^ was used for 1 mL fermentation using 48-well FlowerPlates. Cultures were initiated at an optical density of 0.05 (OD_600_) in an SC medium containing 2% glucose without leucine and incubated for 24 h in triplicate.

For 250 mL shake-flasks cultures, β-carotene-producing yeast (harboring *CrtI*, *CrtE*, and *CrtYB* genes) were cultured in a 25 mL YPD medium with an initial optical density of 0.05 and incubated at 30 °C with 250 rpm agitation for 3 days. For the iron supplementation experiment, iron sulfate was supplemented to a YPD medium with concentrations of 0, 10, 20, 30, 40, and 50 mg/L. Yeast samples were collected at 24, 48, and 72 h for β-carotene extraction, optical density, and yeast biomass quantifications. Similarly, the integrated P2–6 strain (additionally harboring the *EfmvaE*, *EfmvaS*, and *ERG20* genes) was cultured in a 25 mL YPD medium with an initial optical density of 0.05 and incubated at 30 °C with 250 rpm agitation for 3 days.

### β-Carotene Extraction

In a screening of combinatorial production, yeast from a 1 mL culture in the BioLector were vortexed with 0.6 mm glass beads (Sigma-Aldrich, USA) in absolute ethanol for 10 min and chilled on ice for 2 min. These extraction steps were repeated 3 times. The carotenoids in the supernatant from ethanol extraction were directly quantified by high-performance liquid chromatography (HPLC). β-carotene extraction from iron supplementation was performed using the same procedure.

For shake-flask production, carotenoid extraction was performed as previously reported.^8^ Briefly, yeast cells were harvested in 2 mL microcentrifuge tubes and subjected to centrifugation at 12,000 rpm for 3 min. The cell pellets were resuspended in 1.5 mL of 3 N hydrochloric acid and boiled for 5 min. After that, the samples were cooled on ice for 5 min and centrifuged at 12,000 rpm for 3 min. The pellets were washed once with 1.5 mL of deionized water subsequently resuspended in 1.5 mL of dimethyl sulfoxide and vortexed vigorously for 10 min. The extraction solution was then transferred to a 15 mL Falcon tube with the addition of a 2 mL acetone and petroleum ether mixture (ratio of 1:2), followed by rigorous mixing and a 5 min incubation at room temperature. A 4 mL of a 20% sodium chloride solution (w/v) was added and mixed vigorously. To separate the organic phase from the aqueous phase, the mixture was centrifuged at 8,000 rpm for 3 min. The organic phase, which appears at the top of the extraction solution, was subsequently transferred to a light-protected vial and subjected to HPLC.

### High-Performance Liquid Chromatography (HPLC) Analysis

Carotenoid quantification was performed using the HPLC system (Vanquish) equipped with the Hypersil GOLDTM C18 column (4.6 mm × 150 mm) (Thermo Scientific, USA) as previously reported.^8^ The liquid chromatography (LC) program utilized a mobile phase composed of acetonitrile, methanol, and isopropanol in a volumetric ratio of 5:3:2 at a 1.2 mL/min flow rate. The column temperature was maintained at 20 °C. β-carotene was identified using a diode array detector (DAD) set to a visible light detection mode at 450 nm wavelength. In this study, a pharmaceutical secondary standard for β-carotene (PHR1239, Sigma-Aldrich) was used as a reference standard. Additionally, related metabolites such as glucose, pyruvate, acetate, ethanol, and glycerol were quantified using the HPLC system (Vanquish) equipped with a Shodex SH1011 column (8.0 mm × 300 mm) (Resonac, Japan). A 3 mM perchloric acid solution for the mobile phase was maintained at a flow rate of 0.4 mL/min. The column temperature was maintained at 30 °C. Related metabolites were identified using a refractive index detection (RID) system. Welch’s *t* test in R programming was applied to statistically compare β-carotene levels.

### Training and Fine-Tuning the Machine-Learning Model

A gradient boosting algorithm, XGBoost,^44^ was employed in the Learn step during the DBTL cycle. Promoter and terminator strengths determined by GFP intensity measurement were integrated into each combinatorial combination of *EfmvaE*, *EfmvaS*, and *ERG20* genes. Collectively, β-carotene titers from HPLC measurement were together used in machine learning training. Promoter and terminator strengths were centralized and scaled before training the XGBoost model. In the first XGBoost training, data from randomly selected colonies composed of strengths combination and its consequent β-carotene titer were split into 70% training data and 30% test data. A random search strategy was used for hyperparameter tuning. Hyperparameters including the learning rate (eta), maximum tree depth (max.depth), minimum sum of instance weight (min.child weight), training subsample ratio (subsample), and column subsample ratio (colsample by tree) used as described in the previous study.^24^ Each hyperparameter combination was used for XGBoost training with 5-fold cross-validation. Model performance was evaluated by prediction accuracy (*R* ^2^) from the validation of test data, and root-mean-square error (RMSE) of each model training. This workflow was repeated 100 times for unbiased random train-test splitting. The top-performing models were used to predict the remaining combinatorial combinations. The second round of the DBTL cycle followed the same pipeline with increasing input data.

Analysis and machine learning were performed in R programming version 4.1.1.^37^ The XGBoost^45^ and caret^46^ packages were used in machine learning analysis. Visualization and data transformation packages, including ggplot2,^47^ reshape2,^48^ dplyr,^49^ and readxl,^50^ were used in the analysis. The codes are available on the GitHub repository, https://github.com/IMCTlab/ML-beta-carotene-optimization.git

## Results

### Fine-Tuning Expression of the Mevalonate Pathway Using a Promoter-Terminator Library Increased β-Carotene Production

Increasing metabolic flux through precursor pathways is a well-established metabolic engineering strategy. For β-carotene biosynthesis, farnesyl pyrophosphate (FPP) from the MVA pathway serves as the main substrate for carotenogenic enzymes. To enhance FPP production, we fine-tuned the expression of key rate-limiting genes in the MVA pathway: *ERG10*, *ERG13*, *HMGR*, and *ERG20*. Previous studies have highlighted the potential of using *EfmvaE* and *EfmvaS*, two heterologous genes from *E. faecalis* encoding acetyl-CoA thiolase and HMG-CoA reductase, respectively.^51,52^ These genes functionally mimic the native *S. cerevisiae* genes *ERG10*, *ERG13*, and *HMG1/2*, which catalyze the conversion of acetyl-CoA into acetoacetyl-CoA, HMG-CoA, and mevalonate. In this study, we coexpressed native *ERG20* with *EfmvaE* and *EfmvaS* to boost FPP synthesis. To modulate gene expression levels, we constructed a combinatorial promoter and terminator library and used it to fine-tune the expression of these genes in β-carotene–producing yeast (Figure 1A,B).

**Figure 1:**
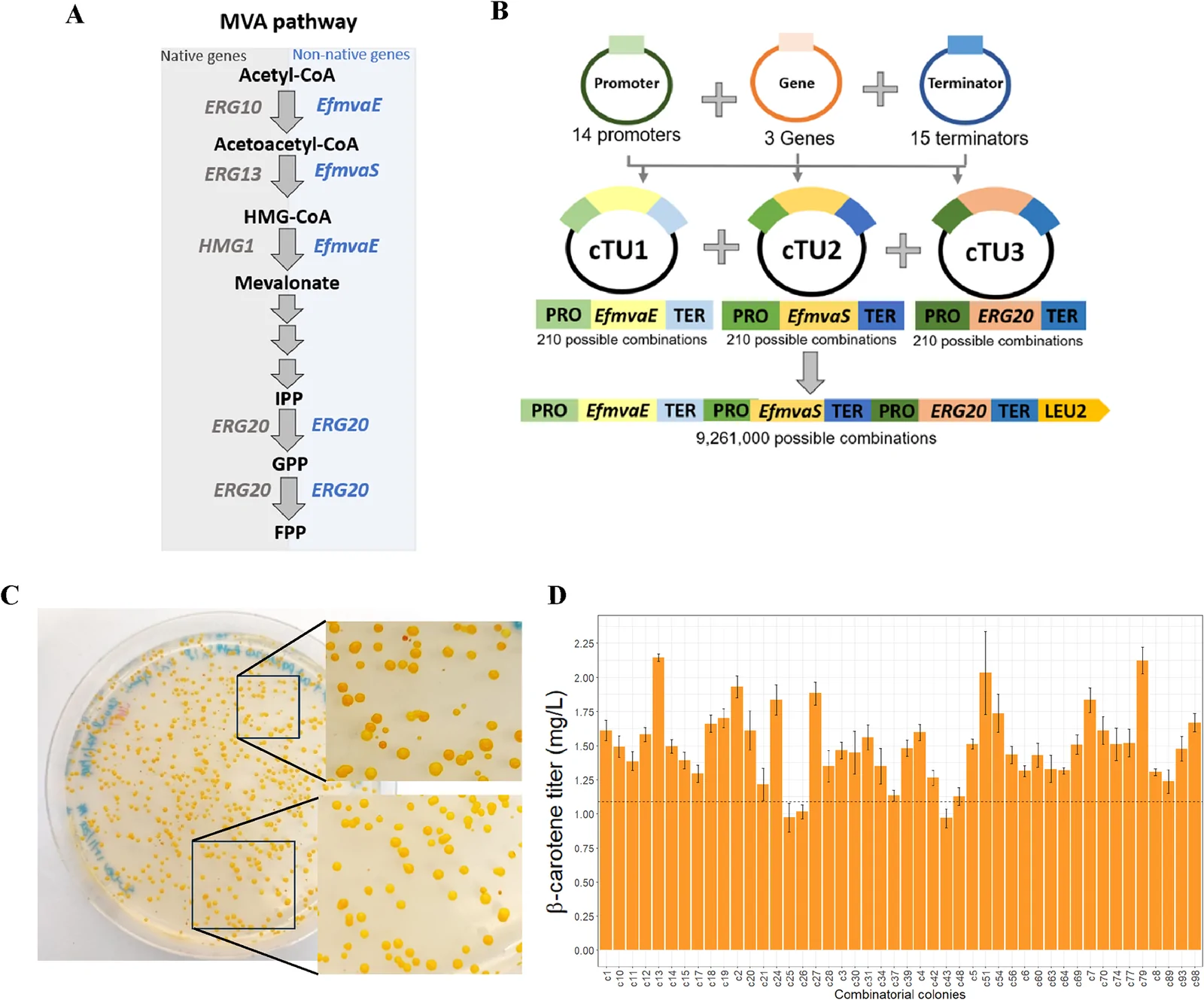
Combinatorial construction of promoter and terminator libraries for fine-tuning MVA gene expression. (A) Rate-limiting MVA genes in *S. cerevisiae*, including *ERG10*, *ERG13*, *HMG1*, and *ERG20* (gray color indicates native genes) compared to heterologous MVA genes from *E. faecalis* (blue color indicates non-native genes; *EfmvaE* and *EfmvaS*). (B) A diagram illustrates the concept of combinatorial library construction using 14 promoters, 3 genes, and 15 terminators. (C) β-carotene-producing colonies harboring rate-limiting MVA genes from transformation of combinatorial plasmids. (D) β-carotene production of 46 selected colonies revealed a broad ranged effect on β-carotene titers. The horizontal dashed line indicates the β-carotene titer of the control strain.

To achieve precise control of expression levels, we selected 14 promoters and 15 terminators based on prior studies investigating transcriptional effects.^53,54^ These regulatory elements were used to construct transcriptional units for *EfmvaE* (cTU1), *EfmvaS* (cTU2), and *ERG20* (cTU3) using the YeastFab system.^42^ The transcriptional units were then assembled into combinatorial constructs via Golden Gate assembly, generating a library of rate-limiting MVA genes with diverse promoter-terminator combinations (Figure 1B). With 9,261,000 theoretical combinations, this library allowed us to explore a wide range of expression levels for rate-limiting enzymes in the MVA pathway. Transforming these combinatorial constructs into β-carotene–producing yeast yielded colonies with varying orange-shaded colors, reflecting differences in β-carotene production (Figure 1C,D). Colonies were intentionally selected based on varying intensities of orange pigmentation on the agar plates to capture a diverse range of β-carotene production levels.

Using the high-throughput BioLector microbioreactor, selected colonies were cultured in triplicate under controlled conditions mimicking a bioreactor. Cultures were initiated at an optical density of 0.05 (OD_600_) and incubated for 24 h. Among the 46 selected colonies, β-carotene production ranged from 0.97 to 2.14 mg/L, while the control strain (without the MVA cassette) produced 1.09 mg/L. Notably, three combinations (c13, c51, and c79) showed an approximately 2-fold increase in β-carotene production compared to the control strain (Figure 1D). Strains exhibiting β-carotene production below the control level may reflect pathway imbalance or metabolic burden caused by excessive expression of an individual rate-limiting gene. For example, strains c26 and c25 contained only the strongest promoters driving *EfmvaE* and *EfmvaS*, respectively. Because these colonies were explicitly chosen from an initial screening pool to maximize phenotypic diversity, their strain identifiers (e.g., c13, c25, c51) are nonsequential. The exact promoter and terminator genotypes for all 46 characterized strains, verified by sequencing, are detailed in Supporting File 2.

These combinatorial constructs resulted in a β-carotene production ranging from an 11.00% decrease to a 96.33% increase relative to the control strain. This demonstrates that the expression levels of rate-limiting MVA genes directly influence FPP production and, consequently, β-carotene biosynthesis. To better understand the metabolic impact, we quantified intermediate metabolites, including glycerol, acetate, and ethanol. However, no consistent correlation was observed between β-carotene levels and these metabolites among the top-producing colonies (c13, c51, c79) (Figure S1).

Therefore, we have demonstrated that fine-tuning gene expression of upstream precursors can have both positive and negative impacts on β-carotene production similar to the direct adjustment of pigment biosynthesis genes.^27^ This result underscores the importance of precise regulatory control in synthetic biology applications.

### Machine Learning Guided by XGBoost Models Accelerates Combinatorial Experiments

Exploring the entire combinatorial space would be impractical due to the significant time and cost required. To address this challenge, we leveraged a computational method, specifically machine learning, to accelerate the process and reduce the number of experiments needed. Instead of relying on trial-and-error approaches with randomly selected combinatorial colonies, we leveraged a Design-Build-Test-Learn (DBTL) cycle integrated with a machine learning framework. (Figure 2A). The 46 diverse strains generated in the previous step were used as a focused training subset, allowing the model to map the sequence-to-function landscape and predict optimal combinations. Although the 46 experimentally characterized strains represent only a small fraction of the 9.26 million theoretical combinations, they were intentionally selected to span a broad range of β-carotene production phenotypes. This sparse but diverse sampling strategy enabled the XGBoost-guided DBTL workflow to identify informative expression patterns without exhaustive experimental screening of the entire combinatorial space.

**Figure 2:**
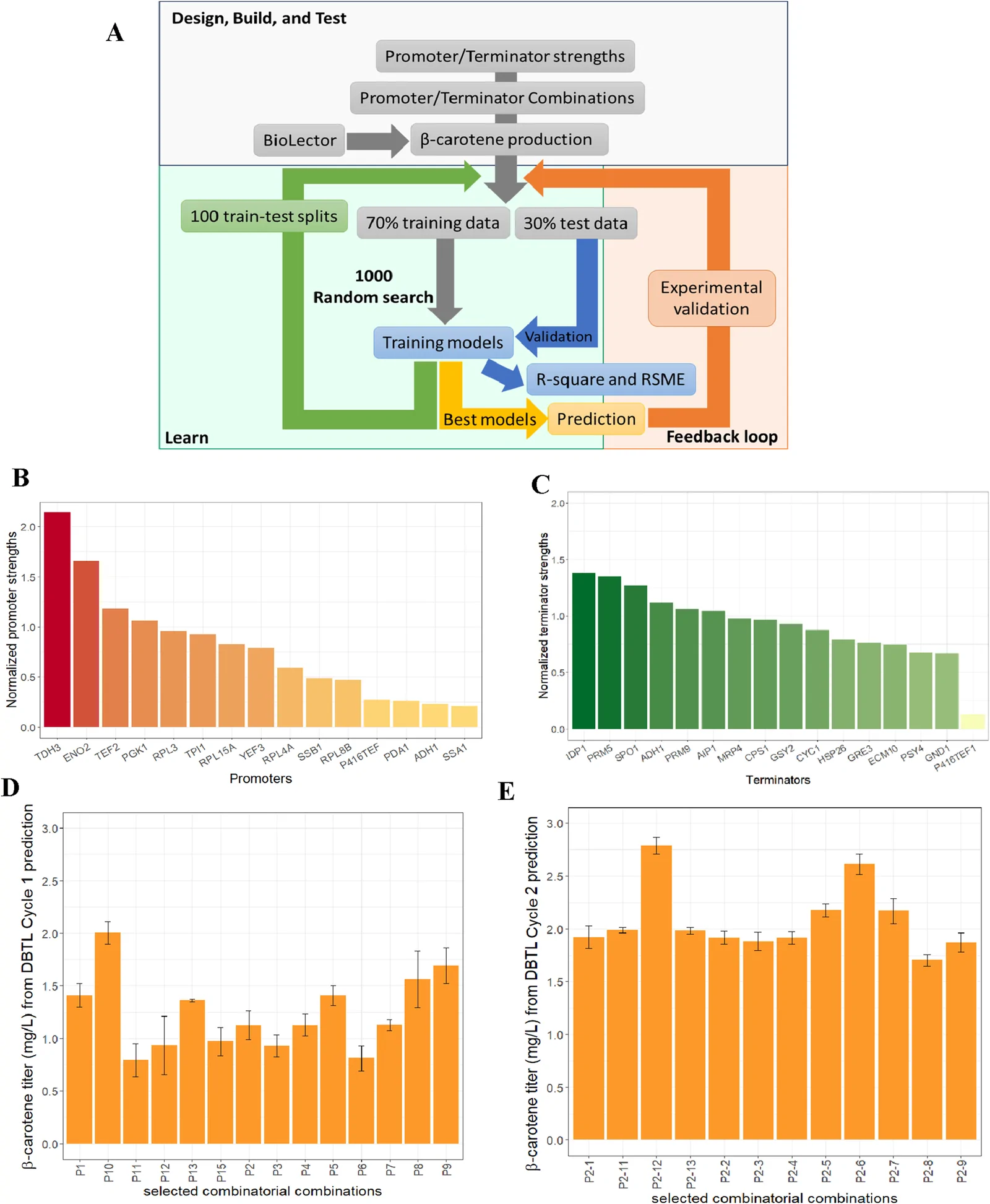
DBTL framework with machine learning (ML) assistance. (A) The Design, Build, and Test phases involved constructing promoter and terminator GFP reporter plasmids to ascertain expression strengths. We then performed combinatorial assembly of rate-limiting MVA genes with promoter-terminator libraries and measured β-carotene production using a high-throughput BioLector microbioreactor. In the Learn phase, XGBoost was applied for model training. The training was repeated 100 times with varying train-test splits (indicated by the green arrow). The top models from 100 train-test splits were selected to predict the remaining combinatorial space, followed by experimental validation. The data obtained from experimental validation was reintegrated into the next Learn phase (Feedback loop) to improve the model training accuracy. (B) Promoter strengths were quantified by GFP intensities and normalized by OD_600_. The normalized GFP intensities were subsequently scaled as inputs for XGBoost training. (C) Terminator strengths were determined using the same procedure as promoter strengths. (D) The β-carotene titers obtained from selected prediction strains in the first round of experimental validation. (E) The β-carotene titers from selected predicted strains in the second round of experimental validation that reintegrated data from the first validation for the XGBoost training.

Before initiating the DBTL cycles, we independently determined the strengths of all 14 promoters and 15 terminators using a GFP reporter assay as shown in Figure 2B,2C. Promoters exhibited more variation in strength compared to terminators. The strongest promoter was TDH3p, followed by ENO2p, TEF2p, PGK1p, RPL3p, TIP1p, RPL15Ap, YEF3p, RPL4Ap, SSB1p, RPL8Bp, PDA1p, ADH1p, and SSA1p. The promoter strengths observed in the β-carotene-producing background were similar to previously reported promoter activities.^53^ For terminators, IDP1t demonstrated the strongest activity, followed by PRM5t, SPO1t, ADH1t, PRM9t, AIP1t, MRP4t, CPS1t, GSY2t, CYC1t, HSP26t, GRE3t, ECM10t, PSY4t, and GND1t. However, our findings differed from earlier reports, where PRM9t and CPS1t were identified as having the highest strengths (Figure 2C).^54^ This variation suggests that differences in background strains may influence gene expression within the cassette. We then incorporated the experimentally determined promoter and terminator strengths into the XGBoost training model alongside combinations from sequencing data and β-carotene production levels.

#### DBTL Cycle 1

The first round of the DBTL cycle utilized the 46 characterized strains as input data. The data on promoter-terminator strength combinations and their corresponding β-carotene levels were divided into 34 training strains (70%) and 12 testing strains (30%) (gray arrow in Figure 2A). The train-test split was repeated randomly 100 times to minimize bias. XGBoost, a gradient boosting algorithm previously demonstrated to perform well in promoter strength prediction,^55^ was employed. To optimize the XGBoost model, we implemented a random search strategy that generated 1,000 hyperparameter combinations. Each combination was evaluated using 5-fold cross-validation. This process was repeated 100 times, and the best models were selected based on their performance (green and yellow arrows in Figure 2A). From the 100 XGBoost models generated during data splitting, we selected the top four models that exhibited the highest *R* ^2^ values and the lowest RMSE for prediction of higher β-carotene production levels (Figure S2 and Table S2). The four best-performing models were then used to predict β-carotene levels for the remaining combinations. The predicted 14 top-performing combinations were selected for experimental validation, prioritizing overlapping predictions across models to reduce errors associated with data splitting. While no new strains in the first DBTL cycle demonstrated higher β-carotene levels compared to the highest β-carotene production from the training data (Figure 2D), they were used to refine the XGBoost training models in the next DBTL cycle.

#### DBTL Cycle 2

To improve prediction accuracy, we reintegrated all validated experimental data from DBTL Cycle 1 through a Feedback loop step (orange arrow in Figure 2A). For the second DBTL cycle, the expanded input dataset (59 total inputs) was then divided into 43 strains for training and 16 for testing. As expected, integrating the validated data from DBTL Cycle 1 substantially improved predictive performance of the models. The strains predicted and experimentally validated in DBTL Cycle 2 achieved β-carotene titers ranging from 1.91 ± 0.06 to 2.61 ± 0.09 mg/L, representing significant improvements of 75.23% to 139.45% compared to the control strain (*P*-value <0.0005). Across all selected strains, the average improvement in β-carotene production was ∼89.90% (∼2.07 mg/L), compared to an improvement of only ∼12.84% in the first DBTL cycle (Figure 2D,E).

The highest *R* ^2^ value from the best model in the second DBTL cycle was 0.7263 and the RMSE was 0.1124 (Table S3). Based on feature importance analysis of the three best models, the promoter of *EfmvaE* demonstrated the lowest contribution to the model’s prediction, while other promoters and terminators contributed proportionally. This finding suggests that *EfmvaS* and *ERG20* expressions may require stronger expression levels compared to *EfmvaE* to enhance β-carotene production (Figure 3C and Table S4).

**Figure 3:**
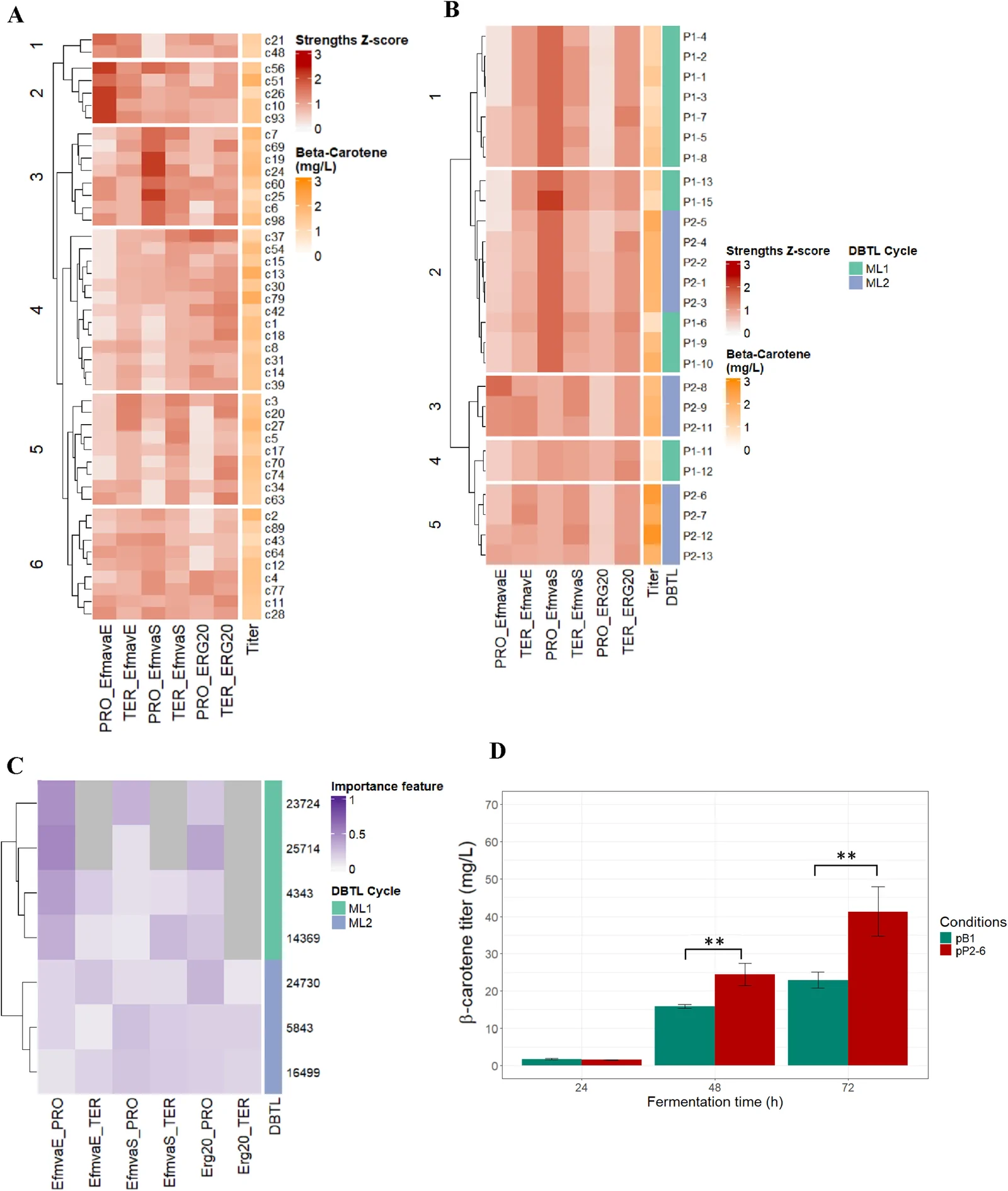
Promoter-terminator combinations and their corresponding β-carotene levels and the selected XGBoost optimized strain compared to a traditional benchmark strain. (A) Clustering of promoter-terminator strengths of the initial 46 strains for the first XGBoost training and (B) Clustering of promoter-terminator strengths derived from validated strains of the first and second DBTL cycles. (C) The distribution of feature importance derived from the top training models of the first and second DBTL cycles. (D) Comparison of β-carotene titers between the pP2–6 strain and the pB1 traditional benchmark strain. Asterisks denote statistically significant differences using Welch’s *t* test. ** indicates *P*-value <0.005.

To visualize the expression landscape and the navigation of the combinatorial space, we constructed a heatmap mapping promoter and terminator strengths to β-carotene levels across the initial 46 training strains and the subsequent predictions validated in DBTL Cycle 1 and DBTL Cycle 2 (Figure 3A,B). As clustering by promoter and terminator strengths, initial 46 strains were grouped based on the presence of a relatively strong promoter for *EfmvaE* in Cluster 2, *EfmvaS* in Cluster 3, and *ERG20* in Cluster 4 (Figure 3A). However, strains with high β-carotene production were distributed across multiple combinations within each cluster, suggesting the presence of underlying regulatory patterns that were not readily identifiable through individual promoter strength alone. Furthermore, to better understand the model’s learning, a feature importance (gain) heatmap was generated to track the contribution of each regulatory element across the four best-performing models in DBTL Cycle 1 and the three best-performing models in DBTL Cycle 2 (Figure 3C). A strong promoter for *EfmvaE* or *EfmvaS* was insufficient to improve β-carotene levels. Promoter-terminator strength combinations associated with high β-carotene levels in DBTL Cycle 2 indicated that fine-tuning expression levels of all rate-limiting MVA genes is more important than strong expression of a single gene (Figure 3B). Feature importance extracted from the best-performing models in DBTL Cycle 2 was also more evenly distributed among the promoters and terminators of *EfmvaE*, *EfmavS*, and *ERG20* than the feature importance from DBTL Cycle 1 (Figure 3C). This analysis revealed dynamic shifts in how the model weighted specific promoters and terminators between cycles, highlighting the value of the feedback loop in refining the predictive rules for optimal pathway balancing.

To experimentally validate whether the XGBoost model effectively learned to balance expression of rate-limiting genes, rather than simply maximizing gene expression in the MVA pathway, we constructed a benchmark strain (pB1). In this strain, all target MVA pathway genes (*EfmvaE*, *EfmvaS*, and *ERG20*) were driven by the strongest promoter (TDH3 promoter) and relatively strong terminators identified in our initial GFP characterization. We compared this overexpressing benchmark against the ML-optimized strain (pP2–6) in a plasmid-based system. The ML-optimized pP2–6 strain significantly outperformed the benchmark pB1 strain, yielding 54.13% and 80.14% higher β-carotene titers at 48 h (*P*-value <0.005) and 72 h (*P*-value <0.005), respectively. Specific β-carotene production was also significantly higher in pP2–6 at 72 h (*P*-value <0.05, Figure S4). These results confirm that simply utilizing the strongest regulatory elements may cause metabolic imbalance or cellular burden, and that the XGBoost model successfully identified the fine-tuned expression levels required to maximize pathway flux.

### Heterologous Expression of β-Carotene-Producing Genes Imposes Metabolic Stress through Iron Deficiency-Related Pathways

To examine the effects of heterologous expression of genes involved in β-carotene production in *S. cerevisiae*, we compared transcriptomes of the β-carotene-producing yeast harboring the *CrtE*, *CrtI*, and *CrtY/B* genes from *S. pararoseus* TBRC-BCC^8^ CX with those of the CEN.PK2–1C parental strain. These genes encode the enzymes of GGPP synthase, phytoene desaturase, and phytoene synthase-lycopene cyclase (a bifunctional enzyme), respectively. These heterologous enzymes utilized farnesyl pyrophosphate (FPP) from the native MVA pathway in yeast, generating a high level of β-carotene, consistent with previous studies.^6,8^

Among genes associated with fundamental metabolic pathways, such as glycolysis, the TCA cycle, gluconeogenesis, and the MVA pathway, we unexpectedly found that only *PGK1*, which encodes a key enzyme in glycolysis, was significantly downregulated (0.8-fold, *P*-value <0.05), while other genes showed no significant changes (Figure 4A,B). This finding suggests that introducing β-carotene production through synthetic biology in yeast does not strongly affect the transcription of key metabolic pathways, including the MVA pathway and overall metabolic flux. Using a log2 fold change cutoff of 0.5 for differentially expressed genes (DEGs), we identified 126 upregulated (log2 fold change >0.5) and 65 downregulated (log2 fold change < −0.5) genes (Figure 4A). Among the most highly upregulated genes (*P*-value <0.0001), *FUR4*, *YDR010C*, and *YPR003C* stood out, with *FUR4* being the only well-characterized gene, functioning as a plasma membrane–localized uracil permease (Figure S5 and Supporting File 4).

**Figure 4:**
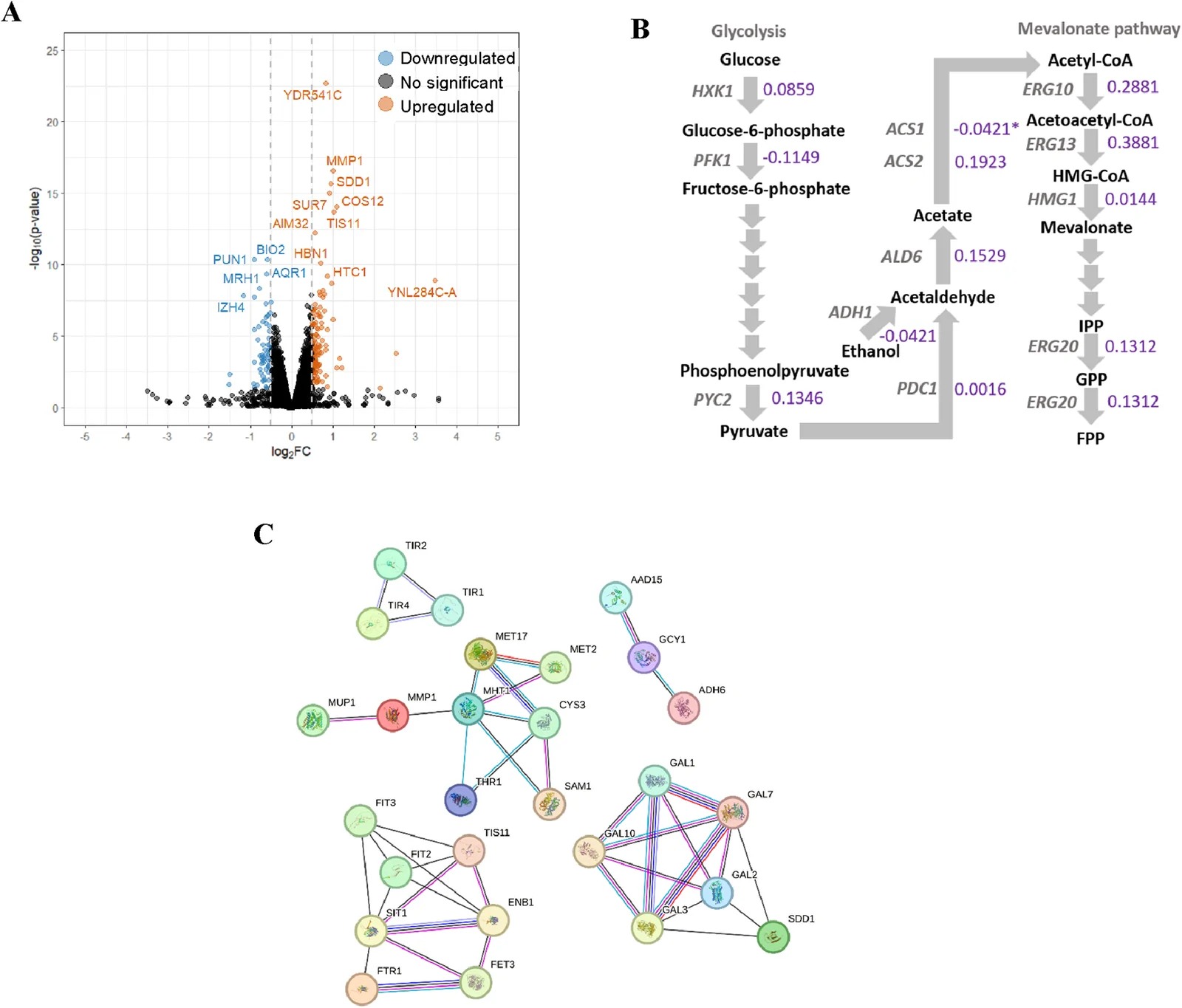
Transcriptomic analysis of the β-carotene-producing yeast compared to CEN.PK2–1C. (A) Volcano plot illustrating differentially expressed genes (DEGs) identified using a log2 fold-change cutoff of 0.5-fold and *P*-value <0.05. (B) Expression changes of rate-limiting genes in the glycolysis and mevalonate pathways. Purple text indicates fold-change values and gray text represents genes encoding key enzymes. The asterisks denote fold-change with *P*-value <0.05. (C) Protein–protein interaction networks derived from upregulated DEGs, highlighting clusters associated with iron deficiency (including iron transporters, iron uptake, and amino acid metabolism) and *GAL* promoter-related genes.

Gene Ontology annotation revealed enrichment for genes involved in general transmembrane transport, sulfur transport, and glutamate dehydrogenase activity, while no significantly enriched functions were observed among the downregulated genes (Figure S6). Protein–protein interaction analysis of the upregulated genes identified three distinct clusters that may have been influenced by the introduction of β-carotene production. Surprisingly, genes associated with iron deficiency were the first cluster identified, including *FIT2*, *FIT3*, *TIS11*, *ENB1*, and *SIT1*, as well as key iron uptake and transporter genes, *FTR1* and *FET3*. The second cluster included genes involved in methionine metabolism (*MMP1*, *MUP1*, *MHT1*, and *SAM1*) and homoserine metabolism (*THR1*, *MET17*, and *MET2*). The final cluster was the *GAL* gene family, which includes *GAL1*, *GAL2*, *GAL3*, *GAL7*, and *GAL10* (Figure 4C).

In response to iron deficiency in *S. cerevisiae*, the transcription factors Aft1/2 activate an iron regulon consisting of approximately 30 genes involved in iron transport and recycling.^56^ We found that the upregulated iron deficiency gene cluster mapped to this iron regulon and partially overlapped with genes known to respond to iron deficiency, such as *MMP1* and *MET2* indicating iron deficiency-like transcriptional response. These findings could serve as a foundation for future β-carotene optimization.

### Combined Optimization of β-Carotene Production by Ml-Assisted Fine-Tuning of MVA Gene Expression with Iron and BHT Supplementation

Having independently explored combinatorial fine-tuning of MVA gene expression and transcriptomic analysis of β-carotene–producing yeast, we next sought to integrate these complementary strategies to further enhance β-carotene production. An investigation of transcriptional alterations in yeast expressing heterologous β-carotene biosynthesis genes revealed an upregulated cluster of iron uptake genes, while no significant changes were observed in the expression of genes associated with key metabolic pathways. These results suggested that β-carotene production may impose a metabolic burden related to iron homeostasis. Building on the upregulation of genes associated with iron deficiency, we explored iron supplementation as a strategy to optimize β-carotene production. To assess its impact, iron sulfate (10–50 mg/L) was added to 25 mL YPD cultures of β-carotene–producing yeast (without the MVA cassette) in 250 mL Erlenmeyer flasks, with three independent replicates per condition. Iron supplementation significantly enhanced β-carotene production, with 10–20 mg/L of iron sulfate yielding the highest improvement, resulting in 80.87% and 70.54% increases in β-carotene titer (14.0 ± 3.26 mg/L and 13.2 ± 2.01 mg/L, respectively) compared to cultures without supplementation (7.74 ± 0.267 mg/L) at 48 h (*P*-value <0.05 in 20 mg/L of iron sulfate supplementation) (Figure 5A). Interestingly, the effect of iron sulfate was time-dependent, showing the most significant impact at 48 h, with reduced enhancement observed at 72 h, and no significant effect at 24 h. Our findings contribute to this growing body of work by highlighting the potential of inorganic supplementation, specifically iron sulfate, as a novel approach to enhance β-carotene production in *S. cerevisiae*.

**Figure 5:**
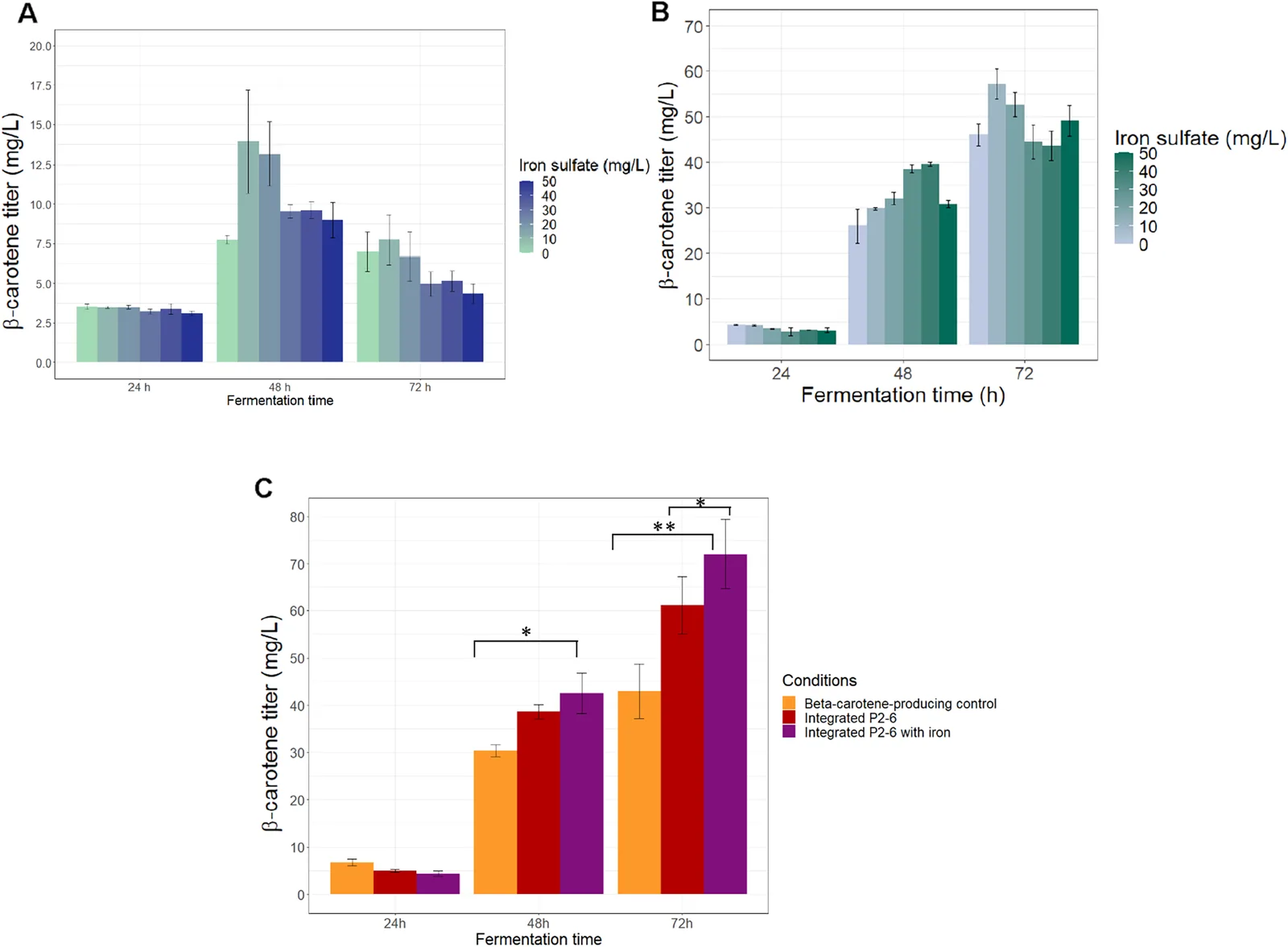
Enhanced β-carotene production through an integrated approach at the 250 mL shake-flask scale. (A) Effect of iron supplementation on the β-carotene-producing yeast from 0 to 50 mg/L of iron sulfate. (B) Effect of iron supplementation with BHT on the β-carotene-producing yeast from 0 to 50 mg/L of iron sulfate. (C) Comparison of β-carotene titers between the integrated P2–6 strain identified in the second DBTL cycle with and without iron supplementation, versus the β-carotene-producing yeast without fine-tuned MVA gene expression. Asterisks denote statistically significant differences using Welch’s *t* test. * indicates *P*-value <0.05 and ** indicates *P*-value <0.005.

Interestingly, a notable decrease in β-carotene titer was observed between 48 and 72 h (Figure 5A). This decline could potentially result from photo-oxidative degradation of carotenoids, as reported in earlier studies showing that β-carotene is highly sensitive to light exposure and oxidative stress.^57,58^ The use of antioxidants such as butylated hydroxytoluene (BHT) has been shown to mitigate pigment degradation in microbial systems.^59^ To evaluate this hypothesis, we supplemented the cultures with BHT. In the presence of 0.5 g/L BHT, β-carotene levels did not decrease at 72 h, confirming that the degradation was primarily an oxidative effect. Under BHT-stabilized conditions, we re-evaluated iron supplementation levels. A concentration of 10 mg/L iron sulfate consistently yielded significantly higher β-carotene titers at 72 h compared to 30 mg/L (*P*-value <0.005) and 50 mg/L (*P*-value <0.05), while showing no significant difference compared with 20 mg/L iron sulfate (*P*-value = 0.1416) (Figure 5A,B). However, from an industrial standpoint, this issue is unlikely to pose a significant limitation because large-scale fermentations are typically performed in opaque stainless-steel bioreactors, which inherently minimize light exposure.

To further investigate the complementary effects of these strategies, we combined iron supplementation with ML-assisted fine-tuning of MVA gene expression. The optimized promoter and terminator combination identified in the P2–6 cassette during the second DBTL cycle was integrated into the genome of the β-carotene–producing yeast strain. Fermentations of the integrated P2–6 strain and the control strain were performed in 250 mL shake flasks containing 25 mL of YPD medium with 10 mg/L of iron sulfate supplementation to evaluate the combined impact of fine-tuned MVA expression and iron supplementation.

At 48 and 72 h, the integrated P2–6 strain both with and without iron supplementation exhibited significant increases in β-carotene titers and specific β-carotene production compared to the control strain (Figure 5C). Specifically, the integrated P2–6 strain with iron supplementation showed a 40.03% increase in β-carotene production at 48 h (from 30.37 ± 1.21 to 42.53 ± 3.32 mg/L compared to β-carotene-producing yeast without integration) and a 67.79% increase at 72 h (from 42.95 ± 5.76 to 72.07 ± 7.36 mg/L) (Figure 5C). The β-carotene titers at 72 h with iron supplementation showed a significant 17.92% increase compared to those without iron addition (72.07 ± 7.36 mg/L vs 61.12 ± 6.11 mg/L) (*P*-value <0.05). The specific β-carotene production was also significantly increased in the integrated P2–6 strain with and without iron supplementation compared to β-carotene-producing yeast without fine-tuned MVA expression. However, no significant difference in specific β-carotene production (mg/g DCW) was observed between cultures with and without iron supplementation at 48 and 72 h (Figure S7).

We then tested the additive impact of iron and BHT supplementation in the integrated P2–6 cultures. This approach resulted in a significant 15.92% increase in titers compared to the control strain with BHT at 48 h and (from 28.83 ± 0.66 to 33.41 ± 1.03 mg/L compared to β-carotene-producing yeast without integration) and a 53.95% increase at 72 h (from 42.54 ± 1.97 to 65.49 ± 3.34 mg/L) (Figure S8). Interestingly, while the combined addition of iron slightly delayed production in the integrated P2–6 strain at 48 h compared to the BHT-only condition (*P*-value <0.001), production in the iron-supplemented cultures recovered by 72 h, showing no significant difference from the BHT-only condition. However, β-carotene titers of integrated P2–6 strain with the BHT-only condition were significantly higher at 48 h (*P*-value <0.005), and no significant additive effect was observed at 72 h These results suggest that iron and BHT supplementation might function through overlapping mechanisms of oxidative protection to improve β-carotene levels. The β-carotene titers of the integrated P2–6 strain supplemented with iron alone were not significantly different from those observed with the combined BHT and iron treatment at 72 h (Figures 5C and S8). This suggests that both strategies converge upon a maximum physiological threshold for β-carotene production in our host strain.

These findings demonstrated the effectiveness of distinct optimization approaches, including ML-guided fine-tuning of MVA gene expression, iron supplementation, and oxidative protection, in enhancing non-native β-carotene production in *S. cerevisiae*. Moreover, combining these strategies with optimization of fermentation parameters may further maximize β-carotene yields in industrial-scale applications.

## Discussion

In this study, we fine-tuned the expression of three rate-limiting MVA genes—two heterologous genes from *E. faecalis* that functionally substitute for three native yeast enzymes, and one homologous gene from *S. cerevisiae* *—*to improve β-carotene production in the engineered carotenogenic yeast. Our findings on individual rate-limiting MVA gene expression^17^ and metabolic flux align with prior studies in *E. coli* production systems, where fine-tuning MVA genes enhanced isoprenoid production.^60^ Similarly, our results support the hypothesis that balancing FPP flux is critical for optimizing non-native carotenoid production.^21,22^ Additionally, adjustment of rate-limiting MVA expressions can positively or negatively impact the β-carotene levels.

Previous studies have also employed combinatorial libraries and machine learning for metabolic engineering. For example, regression analysis has been used to optimize violacein biosynthetic enzymes,^61^ while ANN ensemble models have guided β-carotene and violacein biosynthesis.^27^ Nonrate-limiting enzymes in the MVA pathway have also been optimized for terpenoid production using combinatorial approaches.^23^ In bacterial systems, machine learning has been used to fine-tune key genes for lycopene production.^28^ XGBoost has been integrated into the DBTL cycle for ethanol optimization through combinatorial promoter alteration.^25^ XGBoost training in the promoter modifications required 31 training data to predict a promoter combination for the ethanol improvement strain in the high temperature culture. In this study, we started the XGBoost integrated DBTL workflow with the minimum experimental data based on the previous work.^25^ The iterative feedback loop step has proven effective in enhancing model performance, as observed in previous studies using ANN training for violacein biosynthesis^27^ and ensemble methods for dodecanol production using ribosomal binding site libraries.^29^ This principle is a part of reinforcement learning which learns from previous experimental data as demonstrated in optimization of enzyme levels for improving production.^62^ These findings align with our results, which gained experimental validation of the first DBTL cycle for the next training data in the second DBTL cycle, significantly achieved a 139.45% improvement in β-carotene titers. Additionally, XGBoost model has shown superior predictive performance in scenarios with limited data.^25,63^ The inclusion of low-producing variants alongside high-producing strains is a robust strategy to improve machine learning predictive performance, as demonstrated in the optimization of *p*-coumaric acid production in yeast.^64^ Although the initial training dataset in the present study was primarily composed of β-carotene-producing strains, several strains validated during the first ML prediction exhibited lower β-carotene production than the control. Through our iterative DBTL feedback loop, these data were incorporated into the subsequent training set. By capturing this phenotypic diversity, the model was better able to distinguish between high- and low-performing promoter-terminator combinations. The XGBoost, in particular, provided feature importance that offered insightful information on MVA gene contributions to β-carotene production. Our observed feature importance aligns with the previous research indicating that high expression of *tHMG1*, *ERG19*, and *ERG20* significantly increases β-carotene production.^22^ To our knowledge, this study is the first to demonstrate the potential of XGBoost in optimizing complex promoter-terminator combinations to fine-tune rate-limiting MVA gene expression, demonstrating the potential of complete transcription units for optimization. Moreover, we demonstrated that the combined workflow of XGBoost as a machine learning algorithm with the DBTL cycle from metabolic engineering and synthetic biology approach^65,66^ can be applied with limited training data with a feedback loop for the iteration improvement.

Interestingly, *BIO2*, which encodes an Fe/S cluster protein, was the only consistently downregulated gene during iron deficiency, aligning with previous transcriptomic and proteomic studies. Whereas other reported genes, such as *LEU1*, *ACO1*, *ACO2*, and *ILV3*, showed no significant changes in our dataset.^67,68^ These results suggest that the upregulated genes in the β-carotene-producing yeast are likely related to metabolic stress caused by iron deficiency, with limited overlap with previous studies on physical iron deficiency responses.^67^ These findings could serve as a foundation for future β-carotene optimization. Additionally, the upregulation of the *GAL* gene cluster may reflect the use of GAL promoters in the heterologous expression cassette of the *CrtE*, *CrtI*, and *CrtY/B* genes. To the best of our knowledge, this study is the first to report that iron supplementation can enhance β-carotene production in yeast. The use of iron and iron sulfate as fermentation media components has been reported in several studies.^8,69,70^ For example, a study on yeast growth and iron uptake demonstrated that ferrous sulfate concentrations between 15 and 25 mg Fe/L facilitated efficient iron uptake without inhibiting the growth of *S. cerevisiae*.^71^ In yeast, iron deficiency inhibits ergosterol biosynthesis, which is one of the final products of the mevalonate pathway.^68^ A reduction in farnesyl pyrophosphate (FPP), a key substrate in the MVA pathway, due to its diversion toward carotenogenic biosynthesis, may lower ergosterol levels and thereby mimic the effects of iron deficiency. This metabolic alteration could trigger gene expression changes partially resembling an iron-deficiency response, as observed in this study. Another study reported that iron supplementation increased isobutanol production, suggesting that coordination between mitochondrial metabolism and iron regulation may enhance metabolite production.^72^ We therefore suggest that further investigation into mitochondrial and lipid biosynthetic pathways could provide insights into how heterologous carotenoid expression interacts with iron metabolism. In addition to inorganic supplementation strategies, novel genetic targets that are unrelated to the isoprenoid pathway, identified through transcriptome analysis, have also shown promise for optimization. For instance, overexpression of the *DID2* gene, which functions in the vacuolar protein-sorting pathway, has been reported to increase β-carotene production in *S. cerevisiae*.^73^ Our findings contribute to this growing body of work by highlighting the potential of inorganic supplementation, specifically iron sulfate, as a novel approach to enhance β-carotene production in *S. cerevisiae*.

Recent metabolic engineering efforts in *S. cerevisiae* have yielded diverse β-carotene titers reflecting differences in pathway design, genetic modifications, and fermentation strategies. Advances in multilayer metabolic rewiring and bioprocess control have enabled titers ranging from several hundred milligrams per liter to over 2 g/L in fed-batch bioreactors. For instance, López et al. reported β-carotene titers and contents of 740 mg/L and 6.9 mg/g DCW, respectively, by co-overexpressing *crtE*, *crtYB*, and *crtI* from *Xanthophyllomyces dendrorhous* together with the endogenous *tHMG1* gene.^74^ Sun et al. further enhanced production to 772.8 mg/L (11.4 mg/g DCW) through adaptive laboratory evolution and multiple genetic modifications that enabled β-carotene synthesis from xylose.^75^ Similarly, Fathi et al. achieved 477.9 mg/L and 46.5 mg/g DCW in a lipid-enriched medium by combining carotenogenic gene expression with lipid metabolism engineering.^13^ The most recent work by Lin et al. reached 352.37 mg/L in shake-flasks and 2.09 g/L in a 5-L fed-batch bioreactor by implementing multilayer pathway optimization, enhancing precursor supply, remodeling lipid droplets, and applying atmospheric and room-temperature plasma (ARTP) mutagenesis to obtain a hyper-producing strain.^76^

In comparison, the present study achieved a β-carotene titer of 72.07 mg/L and a specific yield of 9.38 mg/g DCW in shake-flasks through targeted modulation of only three rate-limiting MVA genes combined with iron supplementation. While the resulting titer is modest compared to multilayered strategies, our findings demonstrate that substantial improvement can be achieved with limited genetic perturbation when guided by machine learning and omics-based insights. Rather than competing with large-scale metabolic rewiring approaches, this workflow complements them by offering a rapid, data-driven route for pathway balancing that can be integrated into more complex strain-optimization frameworks.

## Conclusion

Optimizing β-carotene production in *S. cerevisiae* is crucial for developing valuable biotechnological platforms with industrial potential. In this study, we integrated machine learning-guided combinatorial engineering and transcriptomics to enhance β-carotene biosynthesis. We fine-tuned rate-limiting MVA genes and addressed artificial iron deficiency. Using a Design-Build-Test-Learn (DBTL) approach, we optimized homologous and heterologous gene expression through combinatorial promoter-terminator engineering of *EfmvaE* and *EfmvaS* (from *E. faecalis*) and *ERG20* (*S. cerevisiae*). In the second DBTL cycle, the XGBoost-predicted strains achieved a significant improvement in β-carotene titer. In parallel, transcriptomic analysis of the β-carotene–producing yeast revealed that the mevalonate (MVA) pathway, essential for isoprenoid biosynthesis, remained largely unchanged, whereas iron-related genes were significantly upregulated. Integrating fine-tuned MVA expression with iron supplementation further enhanced β-carotene production, demonstrating a significant synergistic effect. In addition, oxidative protection using BHT further improved β-carotene production, although combined BHT and iron supplementation demonstrated no additive effect. This study underscores the power of XGBoost for predicting complex combinatorial designs with limited data and highlights the integration of ML and omics-based insights for metabolic optimization.

## Supplementary Material





## Notes

Primers used; combinations and metabolites titer; selected combination in the DBTL cycle; differentially expressed genes; β-carotene production at flask-scale (XLSX)
Hyperparameters with performance from the first DBTL cycle (Table S1) and the second DBTL cycle (Table S2); Feature importance (Table S3); Summary of major strains used in this study (Table S4); Titers of related metabolites (Figure S1); Performance of the selected top XGBoost models from the first DBTL cycle (Figure S2); Performance of the selected top XGBoost models from the first DBTL cycle (Figure S3); Specific β-carotene production compare to the pB1 strain (Figure S4); Differentially expressed genes (Figure S5); Gene Ontology (Figure S6); Comparison of specific β-carotene production (Figure S7). Comparison of specific β-carotene production and titers in BHT-stabilized culture and iron supplementation (Figure S8) (PDF)

## Abbreviations

ML: machine-learning
DBTL: design-build-test-learn
MVA: mevalonate
GFP: green fluorescence protein
RMSE: root mean square error
FPP: fanesyl pyrophosphate
OD: optical density
DCW: dry cell weight
