# Supplementary material for: Integrating Machine Learning and Transcriptomics to Enhance β‑Carotene Production in Saccharomyces cerevisiae
Source: ACS Omega. 2026 Aug 11;11(33):50249–63. doi: 10.1021/acsomega.6c06012 (PMC13520018; doi:10.1021/acsomega.6c06012)
Supplement: Supplementary file 1 [file ao6c06012_si_002.pdf]

Supplementary information

Integrating Machine Learning and Transcriptomics to Enhance β-Carotene Production in *Saccharomyces cerevisiae*

Peerapat Khamwachirapithak[1,*], Kittapong Sae-Tang[2], Suriyaporn Bubphasawan[2,3], Jongrak Choompuper[2], Xin-Qing Zhao[4], Verawat Champreda[2], and Weerawat Runguphan[2,*]

[1] Department of Biotechnology, Faculty of Science and Technology, Rangsit Campus, Thammasat University, Phahonyothin Road, Khlong Luang, Pathum Thani, 12120, Thailand.

[2] National Center for Genetic Engineering and Biotechnology (BIOTEC), National Science and Technology Development Agency (NSTDA) 111 Thailand Science Park, Phahonyothin Road, Khlong Nueng, Khlong Luang, Pathum Thani, 12120, Thailand

[3] Industrial Bioprocess Innovation Group, The Eastern Economic Corridor of Innovation, National Science and Technology Development Agency (NSTDA), 333 EECi Headquarters Wang Chan, Rayong 21210 Thailand

[4] State Key Laboratory of Microbial Metabolism, Joint International Research Laboratory of Metabolic & Developmental Sciences, School of Life Sciences and Biotechnology, Shanghai Jiao Tong University, Shanghai, 200240, People's Republic of China

[*] Correspondence: peerakha@tu.ac.th and weerawat.run@biotec.or.th

Table S1. Strains used in this study

Strain	Description	Use
CEN.PK2-1C	*MATa* his3Δ1 leu2−3,112 ura3−52 trp1−289 MAL2−8c SUC2	Parental strain for construction of β-Carotene producing yeast
pP2-6	CEN.PK containing plasmid P2-6 (PRPL4A-EfmvaE-TADH1-PRPL15A-EfmvaS-TADH1-RPL8B-ERG20-TPRM9)	Harboring fine-tuned MVA cassette from the second DBTL
pB1	CEN.PK containing plasmid P2-6 (PTDH3-EfmvaE-TIDP1- PTDH3-EfmvaS-TPRM5-PTDH3-ERG20-TSPO1)	Harboring strong expression of MVA cassette
Beta-carotene producing yeast	CEN.PK2-1C with integration of 1021bup-TPRM9-SpCrtE-PGAL1-PGAL10-SpCrtYB-TGAL10-PGAL7-SpCrtI-TCPS1-1021bdown	Parental strain for fine-tuning MVA rate-limiting genes and control for β-Carotene production
P2-6	Beta-carotene producing yeast with integration of PRPL4A-EfmvaE-TADH1-PRPL15A-EfmvaS-TADH1-RPL8B-ERG20-TPRM9	Harboring fine-tuned MVA cassette for synergistic optimization

Table S2. Hyperparameters with performance from the top 4 selected XGBoost models from the first DBTL cycle.

Seed Number	RMSE	R^2	Iteration	Eta	Maxdepth	Subsample	colbytree	minchidw
4343	0.2250	0.9121	3	0.6	9	0.9	0.9	3
25714	0.2577	0.9032	3	0.7	7	0.9	0.9	10
23724	0.2225	0.8781	5	0.7	8	0.9	0.8	7
14369	0.1535	0.8714	5	0.6	3	0.9	0.7	0

Table S3. Hyperparameters with performance from the top 3 selected XGBoost models from the second DBTL cycle.

Seed Number	RMSE	R^2	Iteration	Eta	Maxdepth	Subsample	colbytree	minchidw
24730	0.0826	0.6597	6	0.7	5	0.8	0.8	0
5843	0.0999	0.6023	12	0.3	10	0.9	0.7	1
16499	0.1124	0.7263	10	0.5	4	0.9	0.9	3

Table S4. Feature importance of best performance models from the first and second DBTL cycle

DBTL	Seed number	EfmvaE_ PRO	EfmvaE_ TER	EfmvaS_ PRO	EfmvaS_ TER	Erg20_ PRO	Erg20_ TER
1	4343	0.4111	0.1906	0.1015	0.1183	0.1782	-
	25714	0.5182	-	0.1022	-	0.3794	-
	23724	0.4761	-	0.3095	-	0.2142	-
	14369	0.3325	0.0938	0.0688	0.2841	0.2206	-
2	24730	0.1526	0.2237	0.1251	0.1205	0.2979	0.0799
	5843	0.1518	0.0586	0.2507	0.1918	0.1736	0.1732
	16499	0.0880	0.1581	0.2250	0.1968	0.1778	0.1540

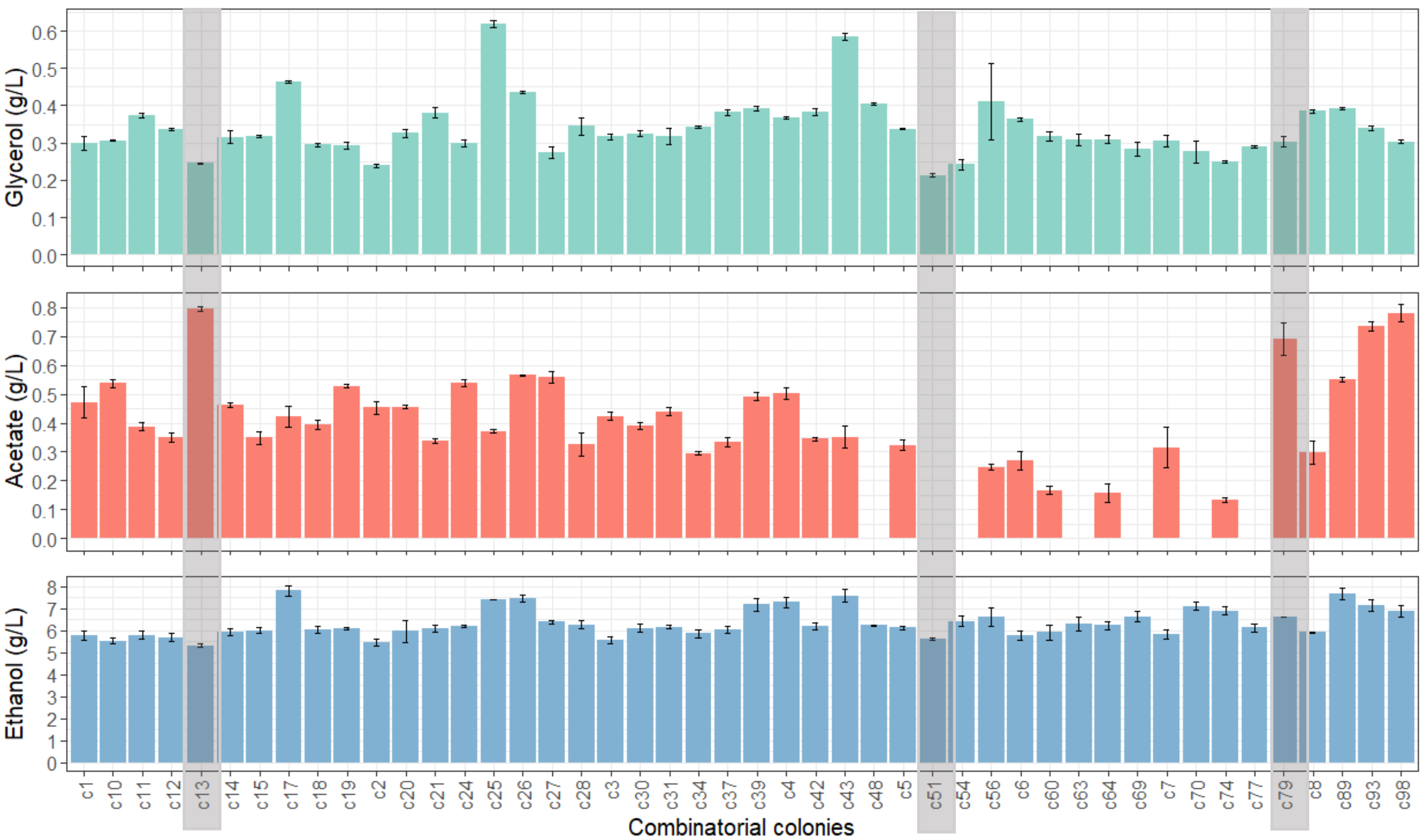

Figure S1. Titers of related metabolites, including glycerol, acetate, and ethanol, from 46 randomly selected colonies from the combinatorial pathway assembly. Grey area indicates a strain combination exhibiting improved β-carotene production.

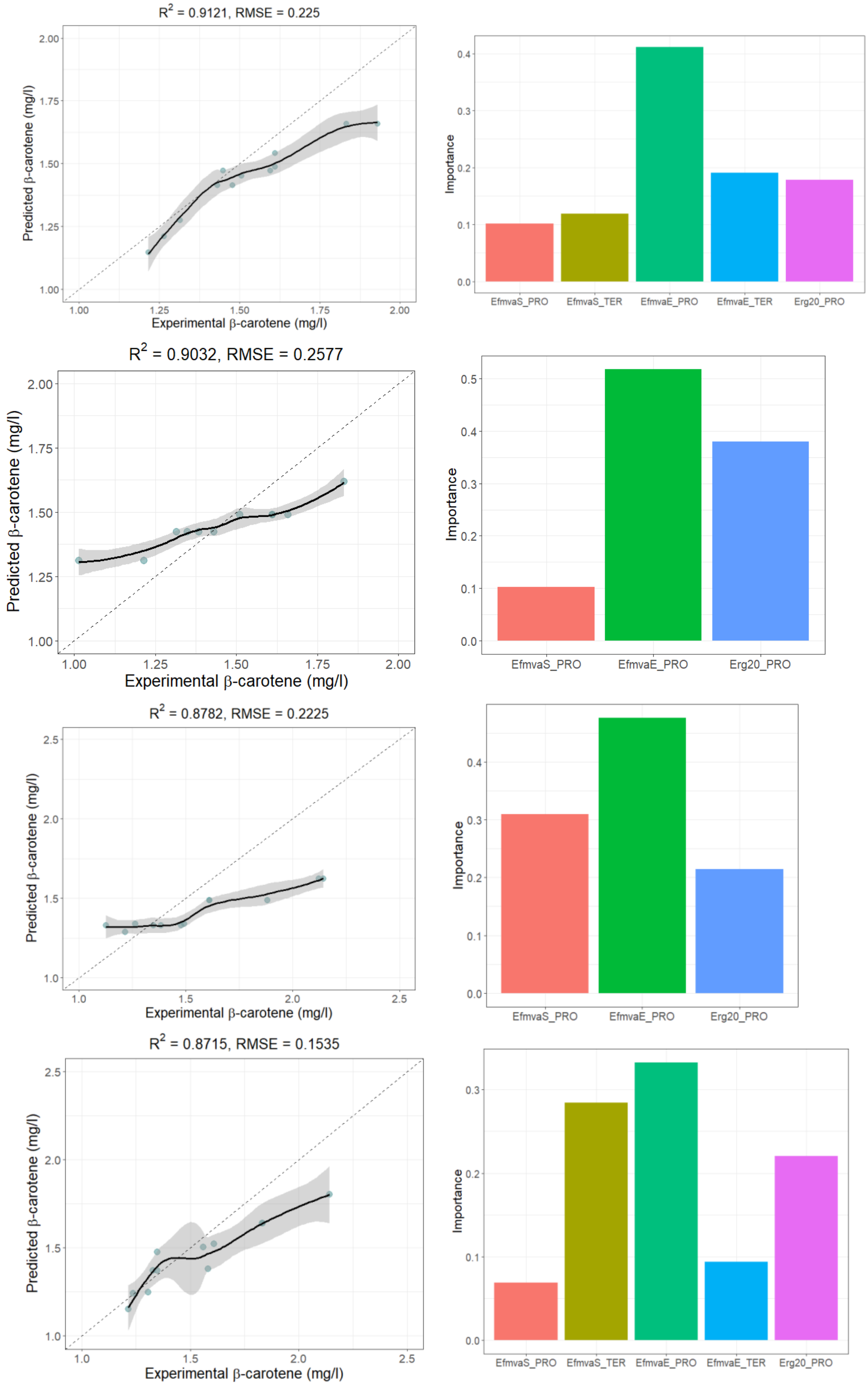

Figure S2. Performance of the selected top XGBoost models from the first DBTL cycle, with their corresponding feature importance.

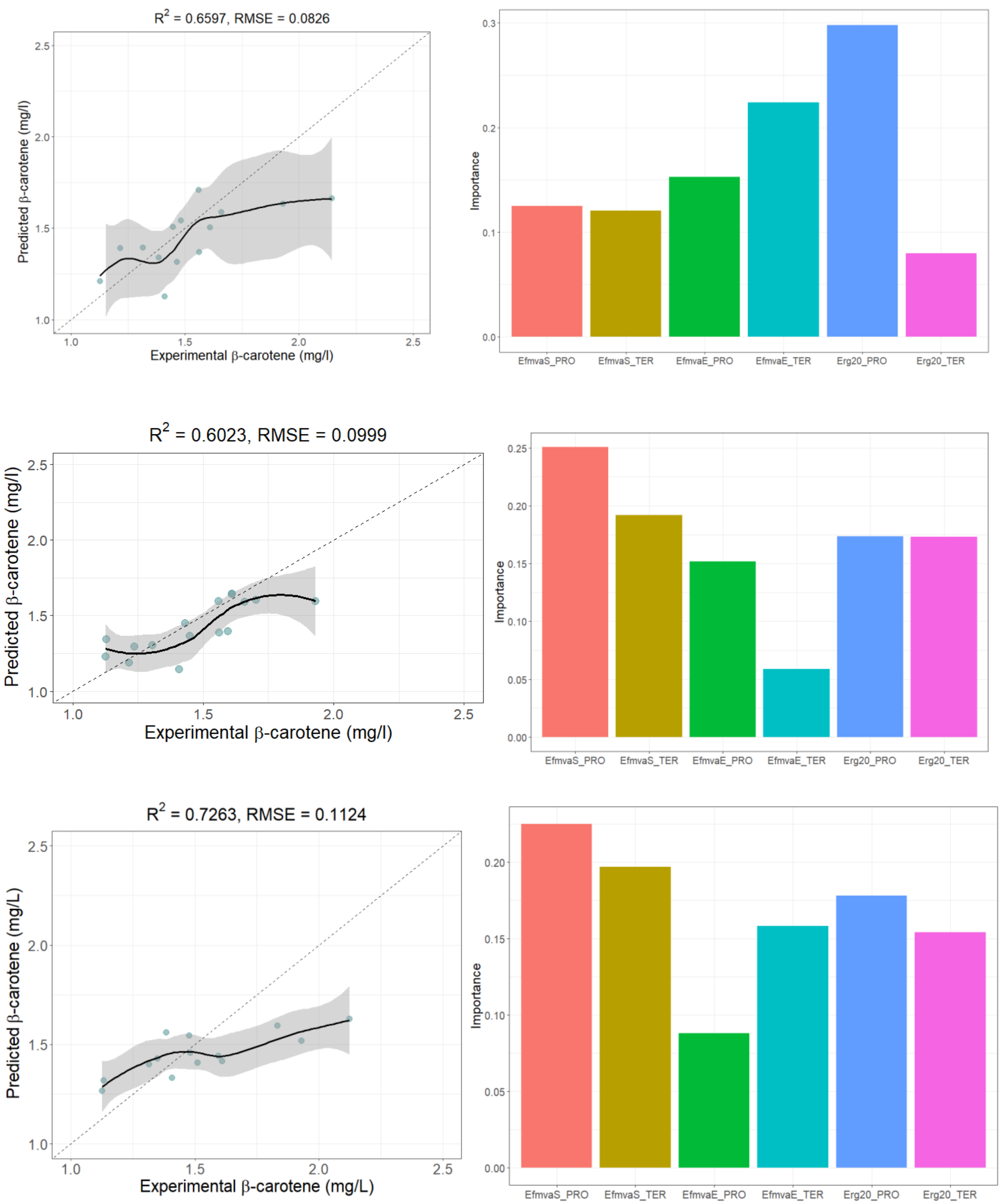

Figure S3. Performance of the selected top XGBoost models from the second DBTL cycle, with their corresponding feature importance.

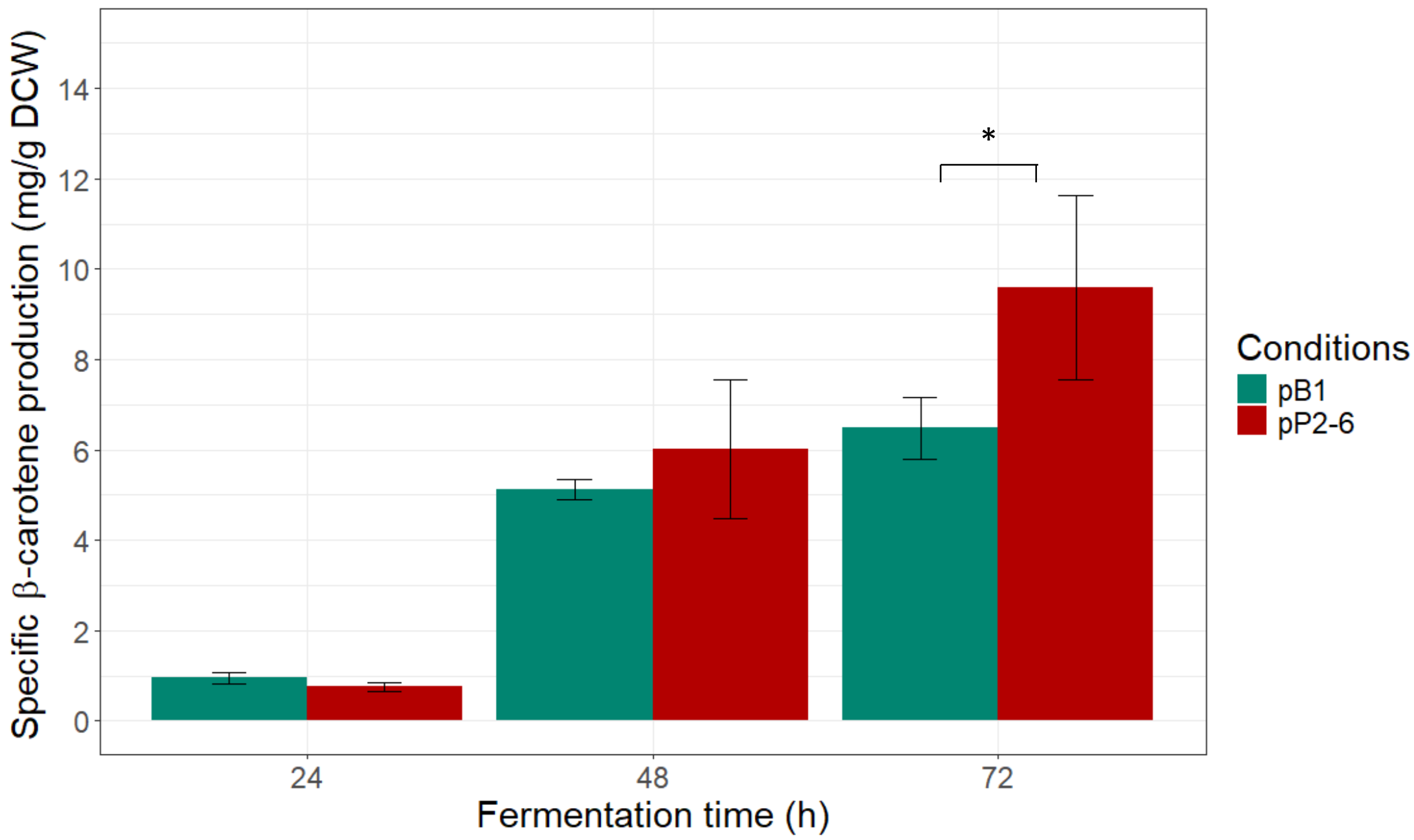

Figure S4. The β-carotene production comparison between the pP2-6 strain and the pB1 strain as a traditional benchmark.

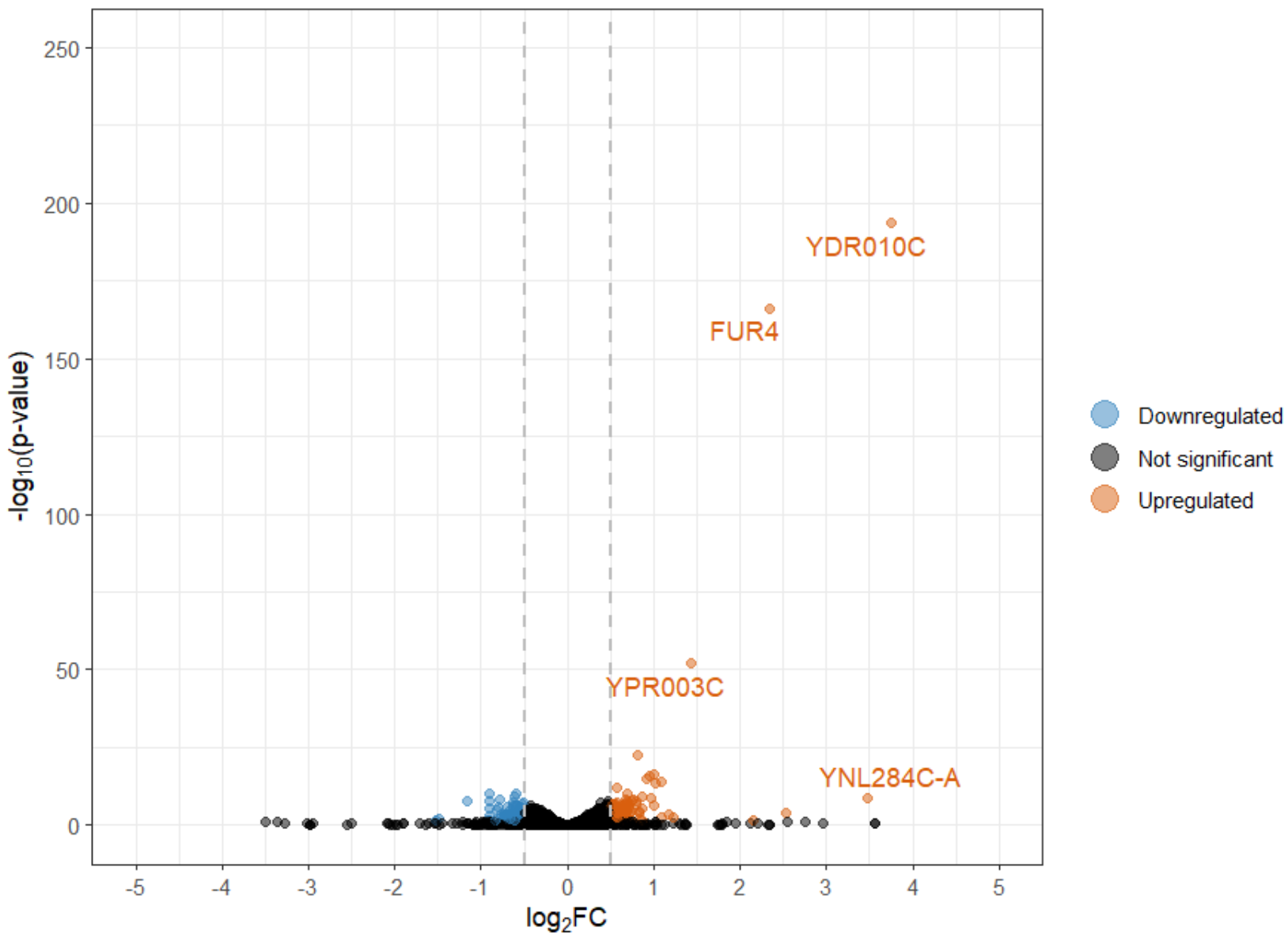

Figure S5. Volcano plot illustrating differentially expressed genes (DEGs) identified using a 0.5-fold cutoff. The y-axis is expanded to 250-fold change for clarity in data representation.

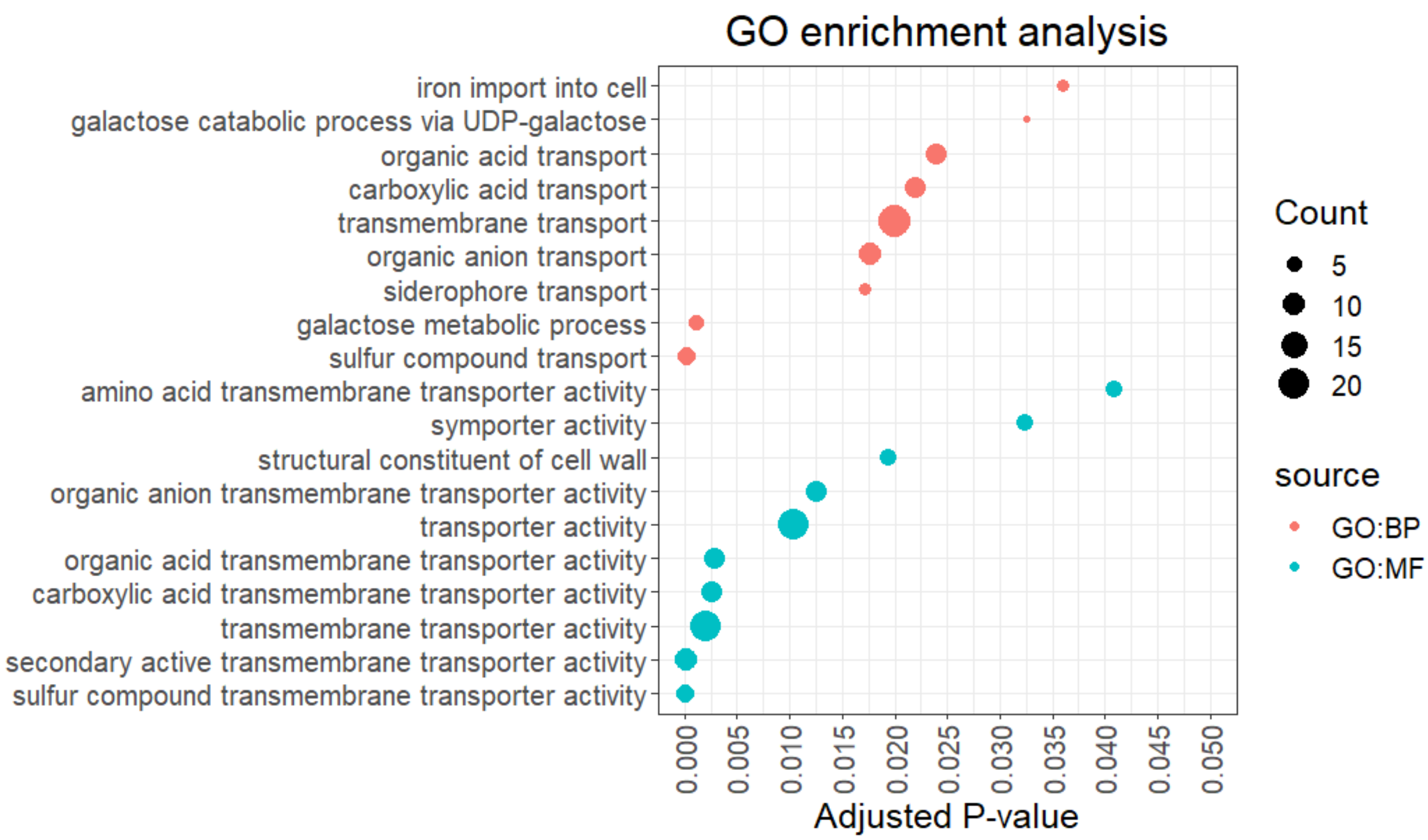

Figure S6. Gene Ontology (GO) annotation from upregulated DEGs

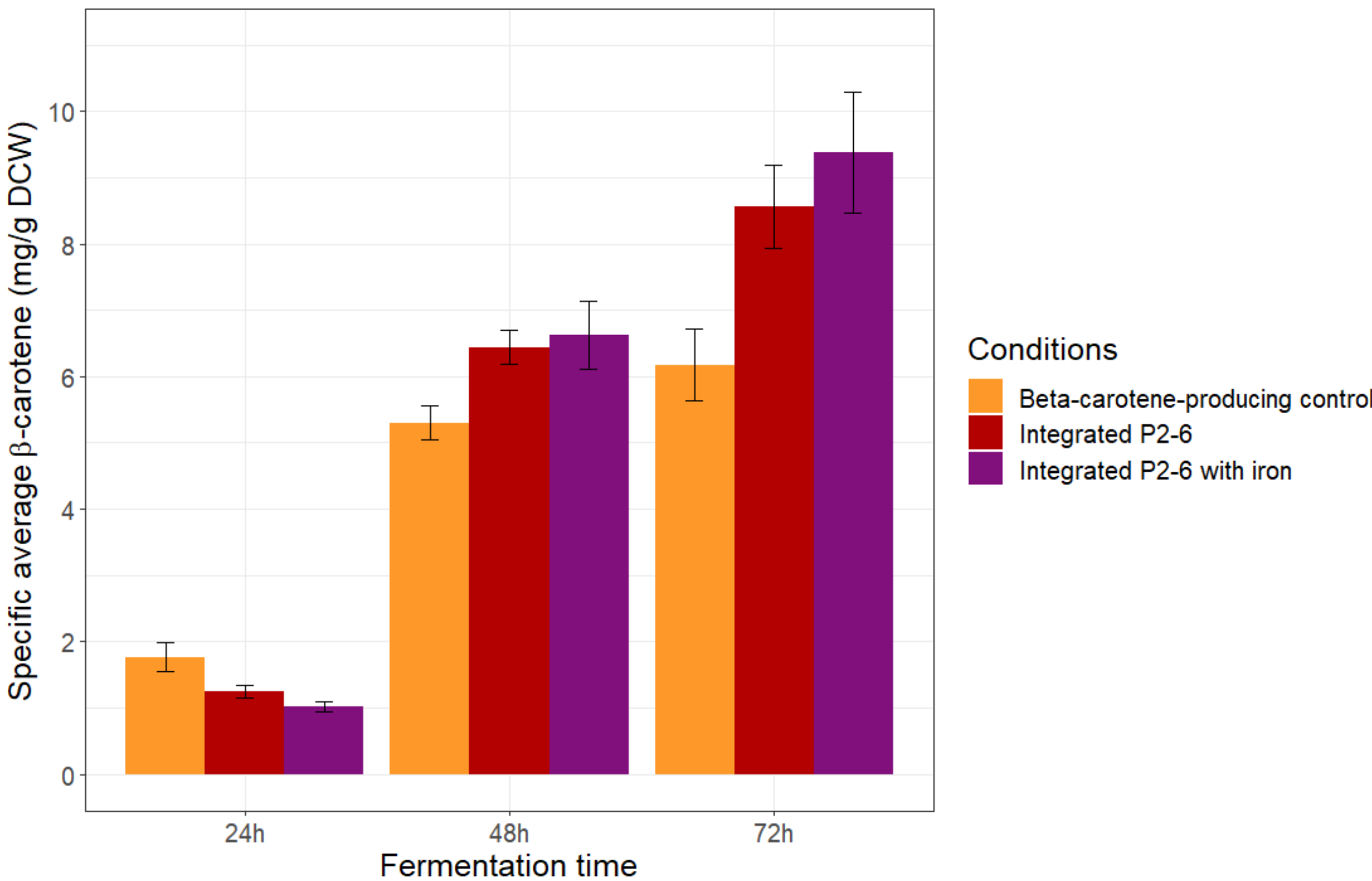

Figure S7. Comparison of specific β-carotene production from the integrated P2-6 combination (identified in the second DBTL cycle) with and without iron supplementation, versus the β-carotene-producing yeast without MVA gene expression fine-tuning.

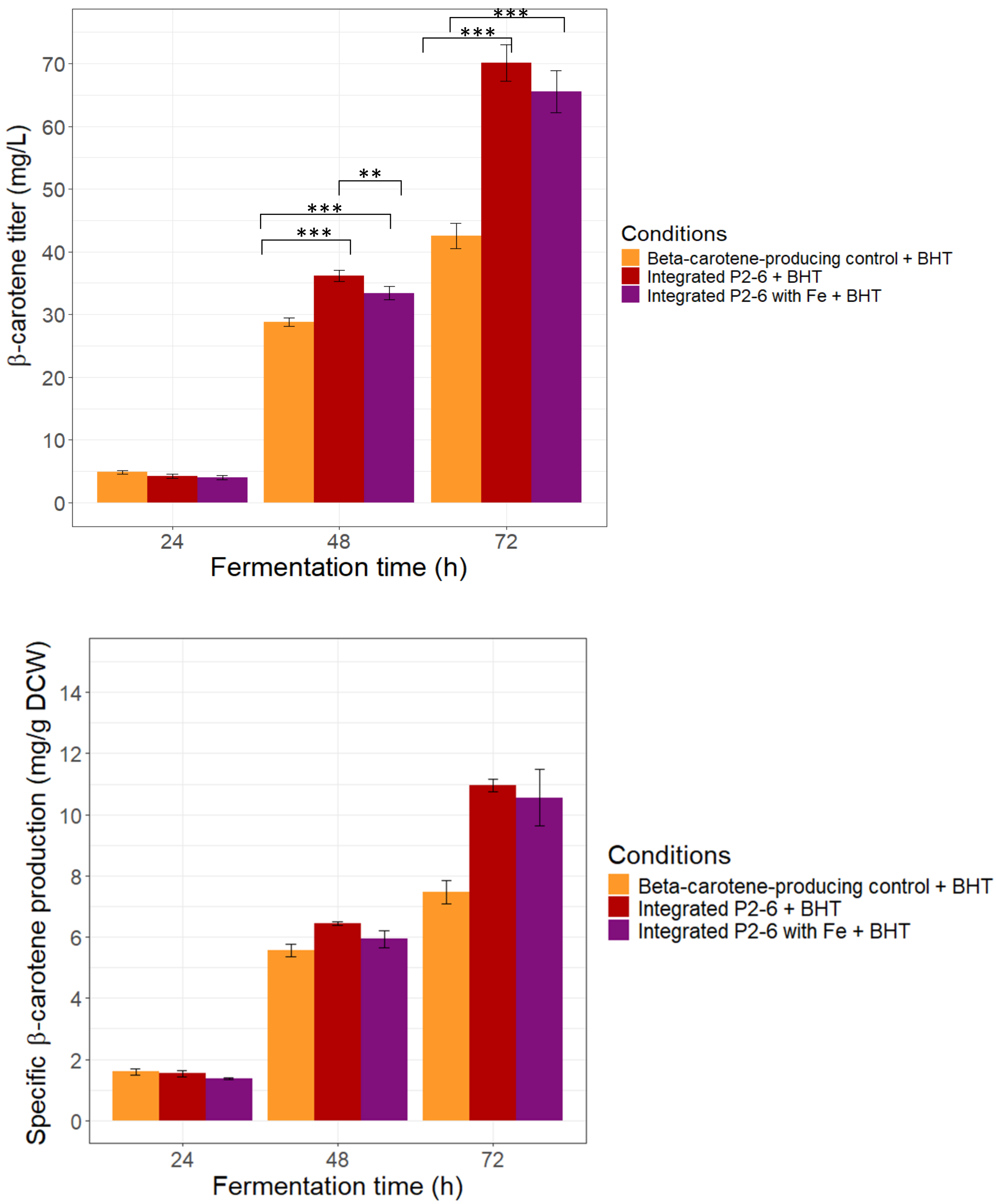

Figure S8. Comparison of specific β-carotene levels from the integrated P2-6 combination (identified in the second DBTL cycle) with and without iron supplementation in the BHT-stabilized culture, versus the β-carotene-producing yeast without MVA gene expression fine-tuning.
